# SnapShot-Seq: A Method for Extracting Genome-Wide, *In Vivo* mRNA Dynamics from a Single Total RNA Sample

**DOI:** 10.1371/journal.pone.0089673

**Published:** 2014-02-26

**Authors:** Jesse M. Gray, David A. Harmin, Sarah A. Boswell, Nicole Cloonan, Thomas E. Mullen, Joseph J. Ling, Nimrod Miller, Scott Kuersten, Yong-Chao Ma, Steven A. McCarroll, Sean M. Grimmond, Michael Springer

**Affiliations:** 1 Department of Genetics, Harvard Medical School, Boston, Massachusetts, United States of America; 2 Department of Neurobiology, Harvard Medical School, Boston, Massachusetts, United States of America; 3 Department of Systems Biology, Harvard Medical School, Boston, Massachusetts, United States of America; 4 Genomic Biology Laboratory, QIMR Berghofer Medical Research Institute, Herston, Queensland, Australia; 5 Departments of Pediatrics, Neurology, and Physiology, Northwestern University Feinberg School of Medicine and Lurie Children's Hospital of Chicago Research Center, Chicago, Illinois, United States of America; 6 Epicentre, Madison, Wisconsin, United States of America; 7 Institute for Cancer Sciences, University of Glasgow, Glasgow, Scotland, United Kingdom; UT MD Anderson Cancer Center, United States of America

## Abstract

mRNA synthesis, processing, and destruction involve a complex series of molecular steps that are incompletely understood. Because the RNA intermediates in each of these steps have finite lifetimes, extensive mechanistic and dynamical information is encoded in total cellular RNA. Here we report the development of SnapShot-Seq, a set of computational methods that allow the determination of *in vivo* rates of pre-mRNA synthesis, splicing, intron degradation, and mRNA decay from a single RNA-Seq snapshot of total cellular RNA. SnapShot-Seq can detect *in vivo* changes in the rates of specific steps of splicing, and it provides genome-wide estimates of pre-mRNA synthesis rates comparable to those obtained via labeling of newly synthesized RNA. We used SnapShot-Seq to investigate the origins of the intrinsic bimodality of metazoan gene expression levels, and our results suggest that this bimodality is partly due to spillover of transcriptional activation from highly expressed genes to their poorly expressed neighbors. SnapShot-Seq dramatically expands the information obtainable from a standard RNA-Seq experiment.

## Introduction

The expression level of an individual mRNA species depends on the rates of key events at three phases of its lifecycle: pre-mRNA transcription, pre-mRNA processing, and mRNA degradation [1]. Each of these steps has the potential to be regulated to control gene expression. Yet the dynamics and regulation of the mRNA lifecycle *in vivo* are poorly characterized, limiting our ability to understand the mechanisms by which gene expression is regulated by chemical compounds, biological signals, or as-yet-uncharacterized RNA-binding proteins, which may number in the thousands [2].

The power of genome-wide approaches in defining key sites of regulation has been demonstrated by recently developed methods for quantifying the *in vivo* occupancy of RNA polymerase across the entire genome [3]–[5]. Building on older single-gene studies [6], these technologies have revealed that transcription is extensively regulated beyond the initial step of RNA polymerase recruitment to promoters [4], [7]. However, the kinetics of other stages of the mRNA lifecycle, such as splicing, are less understood. For example, the estimated time required for splicing ranges from 30 seconds [8]to 12 minutes [9] in studies of individual introns [8]–[12] _ENREF_10. It is unclear whether this variability reflects intron-to-intron variation, species-to-species variation, or the differences in the methods used. The ability to assess *in vivo* splicing rates genome-wide could reveal new modes of gene regulation and identify functions for the many putative splicing factors whose functions remain unknown.

In this study, we describe a new method – SnapShot-Seq – for quantifying mammalian mRNA dynamics, using only standard RNA-Seq data that can be easily generated from any total cellular RNA sample. We introduce a quantitative model that relates the densities of total RNA sequencing (RNA-Seq) reads across exons, introns, and splice junctions to the lifetimes of pre-mRNA intermediate species and mature mRNAs. We describe experimental tests of key assumptions of our method, as well as of its ability to detect specific defects in transcription and splicing. Finally, we apply our method to reveal that the intrinsic bimodality of metazoan gene expression results from a bimodality of mRNA synthesis rates, which in turn may result in part from gene neighbor effects.

## Results

### A quantitative model to measure lifetime from abundance

**Figure 1:**
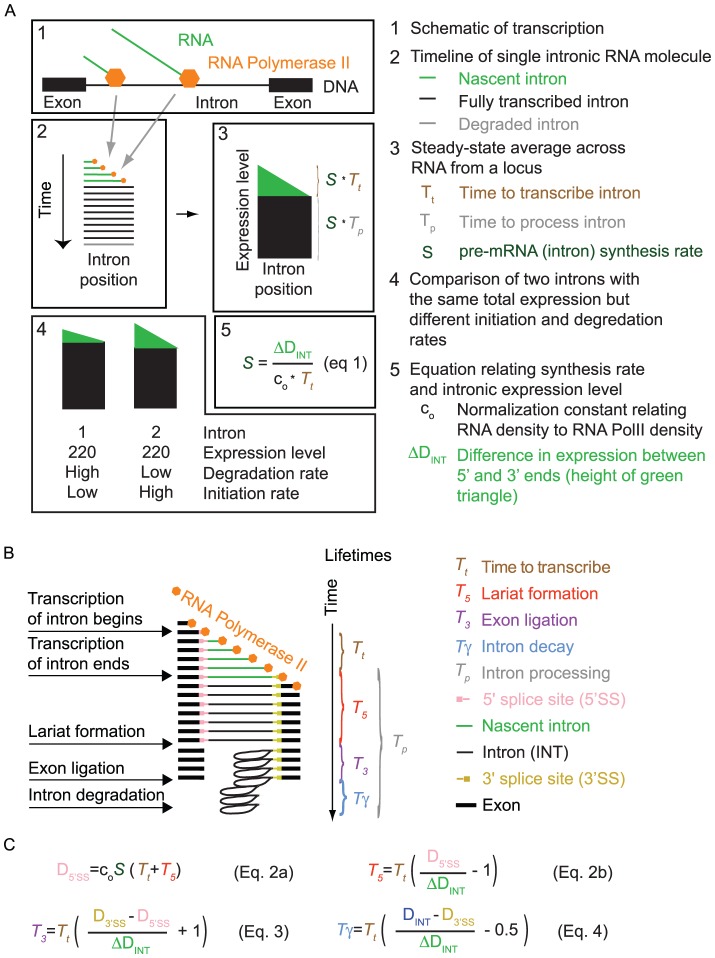
A model for calculating mRNA dynamics from an RNA-Seq snapshot. (**A**) The decrease in expression from 5′ to 3′ along an intron, shown as the height of the green “guillotine” blade, is a product of the rate of intron synthesis (*S*) and the time required to transcribe the intron (*T*_t_). The abundance of the fully transcribed intron at steady state, shown as the height of the black guillotine base, is a product of *S* and the intron processing time (*T*_p_). *T*_p_ consists of the two steps of splicing and intron degradation. Changes in *S* and *T_p_* both affect *total* intron expression level; however, only changes in *S* affect the *difference* in RNA-Seq read density across an intron. The conversion factor *c*_0_ has units of RNA-Seq read density per initiated RNA transcript per cell. (**B**) A detailed timeline of pre-mRNA maturation indicating the four lifetimes (*T*_t_, *T*_5_, *T*_3_, *T*_γ_) that can be inferred from RNA-Seq read densities across three genomic features (INT, 5′SS, 3′SS). (**C**) Equations (2a) – (4) relating the times of lariat formation, exon ligation, and intron degradation to total RNA-Seq read densities. Additional details are provided in the Materials and Methods section.

Our goal was to develop a model that would allow us to simultaneously determine the rates of each step in the mRNA lifecycle solely from a single measurement of total RNA-Seq read densities. A critical first step in this effort was based on the observation that the number of RNA-Seq reads aligning to the 5′ end of an intron is larger than the number of reads aligning to the 3′ end. These decreases in apparent expression level are observed in nascent RNA, nuclear RNA, and total RNA (Fig. 1A) [13]–[16]. We suspected that these decreases could be used to directly infer a gene's rate of pre-mRNA synthesis. This relationship between decreases in expression level along the length of an intron and synthesis rate could then anchor a model for determining the lifetimes of a variety of mRNA intermediates.

We reasoned that the decrease in expression level across an intron should be directly proportional to the number of RNA polymerases transcribing the intron. This proportionality arises because each polymerase actively transcribing the intron has transcribed the 5′ but not the 3′ end of the intron (Fig. 1A). Notably, the decrease in expression level across individual introns does not translate into a decrease in intronic expression level along genes, from one intron to the next. Instead, because introns can be spliced and degraded as soon as they are transcribed, the pattern of intronic expression along genes takes the form of a sawtooth pattern [16]. The pre-mRNA synthesis rate should equal the change in RNA-Seq read density across an intron, divided by the time required to transcribe the intron (*T_t_*, constant for a given intron) and a constant c_0_ that relates read density to transcript number per cell (Fig. 1A, Eq. 1; Materials and Methods).

In relating the pre-mRNA synthesis rate to the rate of intron processing, we found it useful to consider the intronic expression profile as an inverted guillotine blade with a rectangular base. The height of the blade is proportional to the time required to transcribe an intron (*T*_t_; Fig. 1A–B, green) and depends solely on the abundance of nascent introns. The height of the base is proportional to the time required for intron processing (splicing plus intron degradation, *T*_p_; Fig. 1A–B, black) and depends solely on the abundance of completely transcribed (but not yet degraded) introns. The pre-mRNA synthesis rate affects the height of both the base and blade proportionally, and therefore it does not affect the ratio between these two measurements. Thus, the relative times required for intron transcription and intron processing can be inferred from the relative abundances of nascent introns (blade) and completely transcribed introns (base) (Fig. 1A–B).

Building on this framework, we developed a full model relating the times required for transcription and mRNA processing to the abundances of introns and splice sites. For example in Eq. (2a) (Fig. 1C), the 5′ splice site of an intron is created by RNA polymerase as it begins to transcribe the intron at time *t* = 0; the site exists during transcription of the intron (which lasts until time *T*_t_) and persists until the 5′ splice site is cleaved to form the lariat intermediate (which takes an additional time *T*_5_). Thus, the density of RNA-Seq reads across the 5′ splice site (*D*_5′SS_) is proportional to the total duration *T*_t_ + *T*_5_ and the pre-mRNA synthesis rate *S*. To directly solve for the time required for lariat formation (*T_5_*), we can substitute Eq. (1) into Eq. (2a), yielding Eq. (2b). Via a similar procedure, the relationship between RNA-Seq read density and time can be used to infer the times of exon ligation (*T*_3_) and intron degradation (*T*_γ_) (Eqs. 3–4 in Fig. 1C) (Materials and Methods). Given literature values for transcription elongation rate [11], [17], this set of equations can be solved to obtain the times *T*_5_, *T*_3_, *T*_γ_, and the pre-mRNA synthesis rate (a general and detailed treatment appears in Materials and Methods).

### Caveats and limitations to the model

One potential caveat to our model is that the decrease in expression level across introns could be caused in part by exonucleolytic degradation of excised intron lariats (Fig. S1A–D). To address the potential influence of lariat degradation, we compared the decrease in expression level from 5′ to 3′ across introns to the decrease between the 5′ and 3′ splice sites. Both the intron and splice site decreases in expression level should be influenced by the number of polymerases actively transcribing an intron. However, because the splice sites are destroyed during splicing, only the intron decrease should be sensitive to excised lariat degradation (Fig. S1B–D). We performed total RNA-Seq on HeLa cells using strand-specific sequencing of rRNA-depleted total cellular RNA [18], [19]. We observed that the decreases across introns and splice sites were similar, suggesting that exonucleolytic lariat degradation does not contribute to the shape of the intronic expression profile (Fig. S1D–E).

A second caveat is that the intronic expression profile could also be influenced by alternative splice isoforms (*e.g.,* splicing of non-consecutive exons or exon-skipping). To assess this possibility, we quantified the expression levels of alternative splice isoforms. We found that 97–99% of all exon-exon splice junction reads in HeLa cells and mouse neurons were between consecutive annotated exons (Fig. S1F). While apparently surprising, this result is consistent with previous findings: although most genes are alternatively spliced in at least one cell-type or tissue, most exon splicing events do not involve alternative splicing [20]. In addition, alternative splice forms tend to be tissue-specific and expressed at lower levels than constitutive isoforms [21]. Thus on average it appears that the contribution of alternatively spliced forms to the total RNA population is fairly low. Nonetheless, we assessed each of the different classes of alternative mRNA isoforms to determine how each would affect our analysis of the intronic expression profile and found that intronic expression profiles would be minimally affected. (Materials and Methods).

A third caveat is that our model assumes that the rate of transcription elongation is similar among introns and between genes. This is a reasonable assumption on multi-kilobase length scales [5], [11]. On smaller length scales, and in particular near promoters, the effects of pausing can be significant [22], so we included in our analysis only introns larger than 5 kb that start more than 5 kb from the transcription start site. Deviation from linearity will also occur for a short period of time after gene induction [15], [22], but our model is only intended to be applicable under steady-state conditions.

A significant limitation is that although our model should eventually be applicable to individual genes, current datasets do not enable single gene resolution. Instead, to overcome sequence bias and counting noise, both of which can contribute significant error when examining short RNA features such as individual 5′ splice sites (Materials and Methods), it is necessary to average read densities across multiple genes. Given currently available datasets, therefore, our model can be used to produce average processing times, and distributions of these times, for sets of multiple genes.

### Applying SnapShot-Seq to obtain rates of pre-mRNA processing

**Figure 2:**
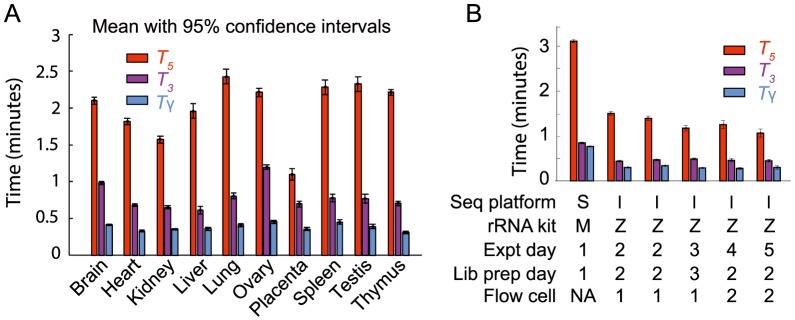
SnapShot-Seq-derived timescales for ten human tissues and technical controls. (**A**) Lifetimes obtained from total RNA-Seq performed on ten human tissues, using the SOLiD Whole Transcriptome Sequencing method [23] with RiboMinus rRNA-depletion. *T*_5_, *T*_3_, and *T*_γ_ are as defined as in Fig. 1. (**B**) A comparison of lifetimes across different sequencing methodologies. We performed sequencing on SOLiD (S) or Illumina (I); hybridization-based rRNA depletion with RiboMinus (M) or RiboZero (Z); and compared RNA samples isolated and prepared into libraries on several different days. We performed Illumina total RNA-Seq using the dUTP method [19]. Error bars indicate 95% confidence from Monte Carlo simulations from individual biological samples.

We used our model to determine mRNA processing times (*T*_5_, *T*_3_, and *T*_γ_, as defined in Fig. 1C) for ten human tissues after performing strand-specific total RNA-Seq [23] from ribosomal RNA-depleted total RNA isolated from each tissue (Fig. 2A). Using a Monte Carlo approach, we repeatedly randomly sampled sets of five genes and solved for the RNA processing times (Materials and Methods), which produced a distribution for each processing time. Across ten human tissues, we find lariat formation (*T*_5_) takes an average of 1–2 minutes, exon ligation (*T*_3_) takes an average of 30–70 seconds, and intron degradation (*T*_γ_) takes an average of 20–30 seconds. These results are consistent with the results of complementary techniques that have examined specific steps in the mRNA lifecycle at small numbers of introns or genes [8], [11], [24]–[26]. We also used our model to calculate mRNA lifetimes (*T_μ_*). Across the same ten human tissues, the average mRNA lifetime varied from just under 1 hour to nearly 4 hours (Table S1 in File S1). These values are similar to the ∼5 hour average mRNA lifetime found by previous studies in mouse and human samples [27].

To address the consistency of SnapShot-Seq across different technological platforms, we performed total RNA sequencing from HeLa cells while varying the library preparation method, the ribosomal RNA removal method, and the sequencing platform. We also addressed biological variability (Fig 2B). HeLa total RNA prepared using the dUTP-RiboZero method on the Illumina platform [19] was consistent between biological samples, experimental days, library preparation batches, and sequencing flow-cells. We found a 2-fold increase in pre-mRNA processing times when we directly compared the Illumina method to a double-stranded ligation, RiboMinus-based method [23] (Whole Transcriptome Sequencing) on the SOLiD platform (Fig. 2B). This difference may reflect differences in the relative biases of these two sample preparation strategies. While the absolute change in rates was two-fold, the relative rate of each of the individual pre-mRNA processing steps was consistent between platforms. To accurately compare samples in subsequent experiments, we only compared samples prepared in the same batch with the same sample preparation pipeline.

Our results represent the first *in vivo* determination of the rates of lariat formation, exon ligation, and excised lariat degradation based on genome-wide data. From our full model, lariat formation is 2–4 times slower than exon ligation and 4–6 times slower than lariat degradation (Fig. 2A,B), suggesting that it is typically the rate-limiting step *in vivo*. Lariat formation is similar to the average time it would take to transcribe the next intron (∼1.5 minutes for a 4.5 kb intron) and faster than the median time required to complete transcription of the gene (∼5 minutes), based on an assumed average elongation rate of 3.6 kb per minute [11]. These results imply that lariat formation frequently occurs before the transcription of the subsequent intron is complete. Thus, alternative splicing events such as exon skipping are likely to require special mechanisms to prevent the conventional splicing of consecutive exons during transcription of a subsequent intron.

### SnapShot-Seq detects a global decrease in the rate of lariat formation upon treatment of cells with the splicing inhibitor isoginkgetin

**Figure 3:**
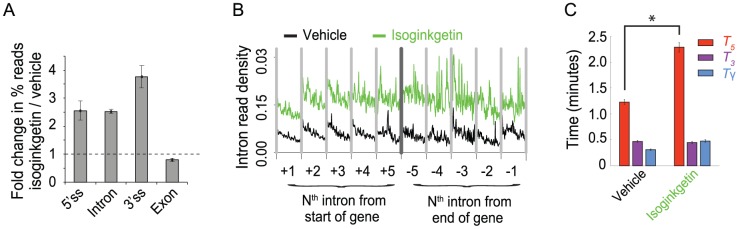
The rate of lariat formation is decreased two-fold by isoginkgetin treatment. (**A**) Genome-wide expression of 5′ splice sites, 3′ splice sites, and introns are increased relative to exons upon treatment of HeLa cells with isoginkgetin (30 µM, 18 hours), based on total RNA-Seq (dUTP method [18], Illumina). The height of each bar indicates the fold change, from vehicle- to isoginkgetin-treated cells, in the mean fraction of reads aligning to each genic feature (*p*<0.02 from two-tailed *t*-tests for all ratios). (**B**) Isoginkgetin treatment increases the “guillotine” base height (*p* = 10^−12^) of intronic expression without increasing the blade height (*p* = 0.5), consistent with a splicing defect (compare to Fig. 1A). Only introns longer than 50 kb from genes with at least 10 introns are included in these meta-intron profiles, which show the last 50 kb of each aggregated intron. Introns of different lengths are aligned at their 3′ ends. RNA-Seq density is normalized as read counts per 10M uniquely aligning reads. The indicated values are from an average of three biological replicates, and *p*-values are from two-tailed *t*-tests based on mean values for aggregated introns 2–10 (n = 9). (**C**) Isoginkgetin treatment leads to a decreased rate of lariat formation (* indicates *p* = 0.02) without affecting exon ligation or excised lariat degradation (*p* = 0.22, 0.08), with calculations as in Fig. 1. *p-*values are from two-tailed *t*-tests with n = 3 biological replicates. Error bars in (A, C) represent s.e.m. from three biological replicates.

To address whether our method could detect specific perturbations in mRNA processing, we treated HeLa cells with the splicing inhibitor isoginkgetin (30 µM), presumed to block splicing by inhibiting the transition from spliceosomal complex A to B (*i.e.,* tri-snRNP binding) [8], [28]. We performed total RNA-seq on three biological replicates of isoginkgetin-treated samples and their paired controls. Consistent with the expectation that isoginkgetin interferes with splicing, RNA-Seq data from isoginkgetin-treated cells showed increased global expression of introns and splice junctions relative to exons (Fig. 3A). The increases were not gene-specific, as most or all expressed genes were affected (Fig. S2A). These results are consistent with isoginkgetin-induced accumulation of unspliced pre-mRNAs.

To measure the decrease in splicing rates caused by isogingketin, we returned to the analysis described above (Fig. 1A). In the intronic expression profile, the guillotine “blade” is derived from nascent introns, and the “base” represents fully transcribed introns that have not yet been degraded. If isoginkgetin were slowing splicing, we should observe an increase in the height of the base. As expected, the height of the base increases ∼2.5-fold with isoginkgetin treatment, indicating a 2.5-fold lower rate of splicing (Fig. 3B). Absent any feedback of splicing inhibition on pre-mRNA synthesis rates, the guillotine blade and intronic slope should remain unchanged. Consistent with these predictions, there are no detectable isoginkgetin-dependent changes in the guillotine blade or intronic slope. Finally, the sawtooth pattern between adjacent introns is still observed in isoginkgetin treated cells, as expected unless splicing were 100% inhibited (Fig. S2B). These results suggest that the intronic expression profile is a useful method for assessing global splicing rates.

Because isoginkgetin is thought to block splicing before its first catalytic step, we tested whether our full kinetic model would detect a specific defect in lariat formation. With isoginkgetin treatment, we observed a nearly two-fold increase in the time required for lariat formation (Fig. 3C, *T*_5_), with no significant effects on exon ligation (Fig. 3C, *T*_3_) or excised lariat degradation. In total, these isoginkgetin-dependent changes in rates would be expected to increase intron processing time (*T_p_*) by ∼2.5-fold, consistent with our conclusions above (Fig. 3B). Our observation, based on *in vivo* genome-wide data, that the rates of lariat formation but not exon ligation are affected by isoginkgetin accords well with the *in vitro* observation that isoginkgetin blocks the formation of spliceosomal complex B.

### Application of SnapShot-Seq to infer genome-wide rates of mRNA synthesis and decay

As discussed above, current sequencing methodologies do not support the application of our full model to individual genes. However, we explored whether a simplified version of our model, based on total RNA-Seq read densities in introns, could be immediately useful for analysis of the synthesis and degradation rates of individual mRNAs. One way to simplify the model might be to use intron read density (D_INT_) as a proxy for pre-mRNA synthesis rate and to calculate the degradation rate by dividing the mRNA abundance by the synthesis rate. The assumption that D_INT_ can act as a proxy for synthesis rate has been made before [29], but has not been validated theoretically or experimentally.

**Figure 4:**
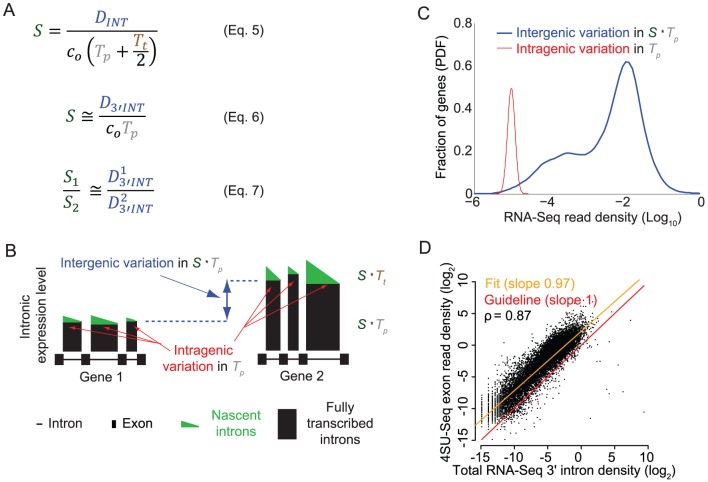
Average expression of the 3′ ends of introns across a gene is an accurate measure of mRNA synthesis rate. (**A**) Equations relating the mRNA synthesis rate *S* to RNA-Seq density across introns (D_INT_) or across the 3′ ends of introns (D_3′INT_). *T_p_* is intron processing time, and c_0_ is a constant relating RNA-Seq read density to transcript number per cell. In Eq. (7), subscripts 1-2 and superscripts 1-2 refer to separate genes. (**B**) The expression levels of the 3′ ends of introns are a useful proxy for mRNA synthesis rates, assuming that the variation in intron processing times among introns is smaller than the variation in mRNA synthesis rates among genes. The schematic shows the contributions of mRNA synthesis and intron processing to expression at the 3′ ends of introns across two hypothetical genes, each with three introns. The second gene is transcribed at a higher rate. (**C**) The assumption stated in (B) holds true: the within-gene standard error of intron densities at the 3′ ends of the (D_3′INT_, red) is much smaller than the range of average D_3′INT_ among genes (blue). For clarity, the distribution of standard errors of D_3′INT_ is shown for the subset of genes with mean intron log-densities within 10% of -5 on the *x-*axis. Data is from mouse neuron RNA-Seq using SOLiD. (**D**) Quantification of mRNA synthesis using RNA labeling with 4-thiouridine (4SU, vertical axis) versus total RNA-Seq (D_3′INT_, horizontal axis). The two methods are correlated with a Spearman's ρ of 0.87. Each point represents one gene and is an average of three total RNA-Seq and three 4SU RNA-Seq samples (biological replicates) from a lymphocyte cell line. Cells were exposed to 4SU for five minutes before cell lysis. Sequencing was performed using the dUTP/Illumina method (total RNA) or standard Illumina RNA-Seq (4SU).

As seen in Eq. (5) (Fig 4A), a caveat to using D_INT_ to estimate mRNA synthesis rate is that intron expression is dependent not only on the mRNA synthesis rate but also on the intron processing time (*T_p_*) and intron length (which affects the intron transcription time, *T_t_*). Using D_INT_ as a proxy for synthesis rates would therefore introduce a significant bias. Specifically, this bias could result in an artifactual correlation between gene length and synthesis rate [30], since longer genes tend to have longer introns, and the read density of long introns is inflated by the longer time it takes to transcribe the intron. To avoid this bias, we estimated the synthesis rate of each gene using the average total RNA-Seq read density of the 3′-most 10 kb of each of its introns (D_3′INT_, derived from the entire intron for introns shorter than 10 kb). On this 10 kb length scale, the contribution of *T_t_*/2 (∼80 seconds, based on 3.6 kb/min [11]) is less than *T_p_* (∼3.5 minutes, sum of times from Fig. 2A), and the influence of intron length is negligible (Fig. 4A, Eq. 6). While sequences shorter than 10 kb could be used to further minimize the contribution of *T_t_*, in this case each intron would be represented by fewer reads. Our strategy balances the benefits of the increased accuracy of quantifying expression using longer sequences while minimizing the length-dependent inflation of intron density.

Using D_3′INT_ as a proxy for pre-mRNA synthesis rate minimizes the bias caused by intron length, but the apparent synthesis rate based on D_3′INT_ still depends on the relative processing times of each intron (*T_p_* in Fig. 4A, Eq 6). Therefore, for this proxy to be useful, the variation in mRNA synthesis rates (*S*) must be much larger than the variation in intron processing times (*T*_p_). A maximal bound on the variation in *T*_p_ can be directly assessed by comparing D_3′INT_ for introns from *the same* gene (Fig. 4B). Intron levels in a single gene are set by *S⋅T_p_*, with *S* constant for all introns synthesized from a common promoter. In contrast, variation in mRNA synthesis rate can be assessed by comparing introns from *different* genes. Variation in intron levels between genes again depends on *S⋅T_p_*, but now *S* is not constant (Fig. 4B). We found that the intergenic variation in D_3′INT_ was at least an order of magnitude larger than the intragenic variation in D_3′INT_ (Fig. 4C). Thus, D_3′INT_ is a reliable proxy for relative mRNA synthesis rate and is influenced comparatively little by intron processing rates (Fig. 4A, Eq 7). This method is practical for single gene measurements because D_3′INT_ is easy to measure accurately using total RNA-Seq. In further support of our analysis, we found that D_3′INT_ is directly proportional to intronic slope, another measure of synthesis rate (Figs. S3A,B).

To independently assess the accuracy of using D_3′INT_ as a proxy for mRNA synthesis rates, we performed sequencing of newly synthesized RNA using 4-thiouridine (4SU) labeling [30]–[32]. We compared our estimate of mRNA synthesis rates based on D_3′INT_ from total RNA-Seq to estimates of mRNA synthesis rates based on quantification of newly synthesized 4SU-labeled RNA. Estimates of synthesis rates from the two methods were linearly correlated (Spearman's ρ = 0.87, Fig. 4D), unlike 4SU-labeled RNA and total RNA-Seq exon densities (Figs. S3C).

**Figure 5:**
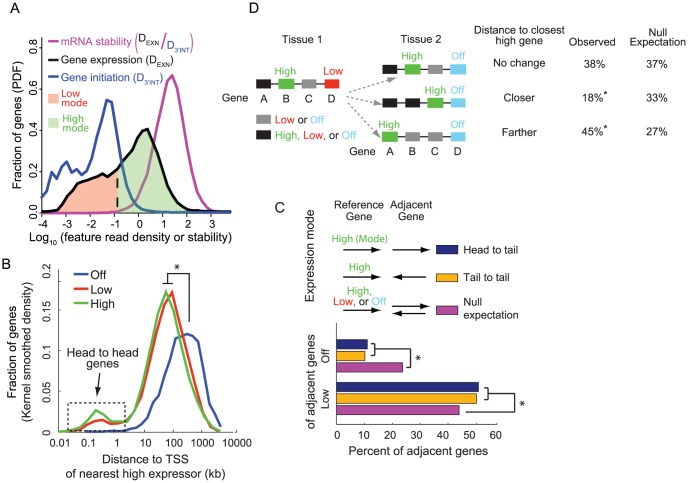
Bimodality of mRNA synthesis rates reflects genome organization. (**A**) Distributions of gene expression levels (D_EXN_), mRNA synthesis rates (D_3′INT_), and mRNA stability (D_EXN_/D_3′INT_) reveal two modes of gene expression. D_EXN_ and D_3′INT_ refer to the RNA-Seq read densities across exons and the 3′-most 10 kb of introns respectively. (**B**) Compared to genes that are not expressed, low and high expressors are found closer to highly expressed genes. The *x*-axis indicates the distance from a gene's transcription start site (TSS) to the TSS of the nearest high expressor; * indicates *p*<10^−3^ by a two-sample K-S test. (**C**) Genes adjacent to high mode genes are disproportionately more likely to be in the low mode and less likely to be in the off mode of gene expression, for both head-to-tail and tail-to-tail gene pair architectures (* indicates *p*<10^−6^ from a bootstrap simulation with one million iterations, in which expression classes were permuted). (**D**) Between tissues, when genes transition from low expressors to non-expressors, their average distance to the nearest high expressor increases (*p*<2.2×10^−16^, based on a chi-square test). The ∼15% difference between the data and the randomized control suggests that at least 15% of the changes from low to off are due to a nearby gene being up-regulated. RNA-Seq data is from mouse cortical neurons sequenced using SOLiD [53] (A–C) and ten human tissues (D).

Together, accurate measurements of mRNA levels and synthesis rates can be used to estimate mRNA lifetimes for individual genes (Fig 5A). Our estimates of mRNA lifetime varied significantly among genes, in agreement with mRNA half-life estimates ranging from 16 minutes to 790 minutes for inducible transcripts [33]. Similarly, high-throughput estimates of mRNA turnover from 4SU-labeled RNA experiments reveal distributions of mRNA turnover rates shaped similarly to our own, with our method having a 5-fold larger full-width at half-max [27]. This larger variance in mRNA lifetimes between genes in our method could result from biological differences, from biases due to the sets of genes examined, or from or the techniques themselves. Overall, these comparisons show that measurements of D_3′INT_ can be a powerful method for extracting mRNA synthesis and decay rates from easily obtainable total RNA-Seq data.

### Bimodality of gene expression reflects genome organization

We applied our ability to assess genome-wide rates of mRNA synthesis and decay to investigate the poorly understood phenomenon of gene expression bimodality. In metazoans, expressed genes fall into one of two categories: a< ∼1 mRNA per cell (low) mode or a > ∼1 mRNA per cell (high) mode [34]. We asked whether this bimodality could be cleanly attributed either to mRNA synthesis or degradation. Using D_3′INT_ to assess synthesis rates, we observed a bimodal distribution of synthesis rates, but not of mRNA stabilities, in a variety of tissues and cells (Figs. 5A, S4A,C–E), strongly suggesting that pre-mRNA synthesis rates are the sole determinant of the observed bimodality. This interpretation is supported by the fact that genes segmented into high and low expression levels are simultaneously segmented into high and low mRNA synthesis rates respectively (Fig. S4B).

We asked whether low mode genes and high mode genes fall into distinct functional categories. Using RNA-Seq data from mouse neurons or HeLa cells, low mode genes specifically are enriched for gene ontology (GO) categories associated with membrane and extracellular compartments (Table S2). These same categories are also enriched among tissue-specific genes (those expressed in only 1-2 of the 10 human tissues we examined). This extensive set of shared categories suggests that the low mode of expression may simply reflect incomplete repression of genes that are not needed in the tissue of question, rather than a need for very low levels of the product of these genes. Supporting this hypothesis, unexpressed genes are similarly enriched for GO categories associated with membrane and extracellular compartments (Table S2).

We therefore sought to identify a mechanism that could explain why some genes are expressed in the low mode while others are not detectably expressed. One cause of the low expression mode could be the presence of nearby genes that are highly expressed. Neighboring genes are more likely to be co-expressed and co-regulated in a variety of organisms including *S. cerevisae* [35]–[38], *C. elegans* [39], and humans [39], [40]. To investigate whether the low expression level of some genes could result from their genomic proximity to highly expressed genes, we examined the distances from genes in the unexpressed, low, and high modes to the nearest high mode gene. We found that low mode genes are far more likely than unexpressed genes to be within 100 kb of a high mode gene (Fig. 5B). This effect occurs both for tail-to-tail and head-to-tail gene pair architectures, indicating that the effect cannot be attributed solely to shared, bidirectional promoters or to transcriptional read-through (Fig. 5C). To evaluate the extent of this potential effect, we considered what happens when a low-expressed gene in one tissue converts to an unexpressed gene in a different tissue. In these cases, the distance to the nearest high-expressed gene increases 45% of the time, compared to a 27% chance expectation (Fig. 5D). The magnitude of this effect suggests the hypothesis that at least 15% of the low expressors are expressed only because of their genomic proximity to high expressors. The strand-independence of these gene neighbor effects suggests that they could be mediated by long-range chromatin interactions, such as DNA looping.

## Discussion

We have developed a new computational method, SnapShot-Seq, for measuring the dynamics of RNA production and processing. The method relies on the fact that the abundances of intermediate RNA species are proportional to their lifetimes. Relying on this proportionality, it is possible using only standard total RNA-Seq data to: derive rates of pre-mRNA synthesis and timescales for specific pre-mRNA processing steps (Fig. 1), detect *in vivo* alterations in the rates of specific steps of splicing (Fig. 3), and obtain genome-wide measurements of mRNA synthesis and degradation rates (Fig. 4). Our approach has several advantages over the existing state-of-the-art methods. First, it requires only standard RNA-Seq data and can thus be performed *post hoc* on existing total RNA-Seq data sets. Second, it does not require any of the perturbations previously needed to determine kinetics of splicing and mRNA degradation, *e.g.,* cellular uptake of a chemical label [41] or interference with RNA polymerase II [11], [42]–[44]. Nor does our method require immunoprecipitation [5] or rely on *in vitro* enzymatic activity [4]. Unlike many competing methods, our method is easily applied to whole tissues, including quantity-limited diseased and normal tissue biopsies from patients. Our method should prove informative for examining splicing rates in diseases – such as retinitis pigmentosa, myelodysplastic syndrome, and lymphocytic leukemia – whose etiologies involve RNA processing defects that remain poorly understood [45]–[48]. Finally, our method is unique in simultaneously assessing many aspects of mRNA dynamics, an advantage that could prove useful in understanding the interconnections among different steps in pre-mRNA processing.

Our current SnapShot-Seq analyses rely in many cases on average abundances across multiple introns in order to precisely compute the intron slopes and splice site read densities that are crucial inputs to our dynamical model. As sequencing technologies and library preparation methods improve, SnapShot-Seq will allow the dynamics of each step of splicing to be precisely determined for individual genes and individual introns (Materials and Methods). Eventually, increased sequencing read depth and reduced bias should provide accurate read densities at single-nucleotide resolution, making it possible to extend our method to measure nucleotide-by-nucleotide RNA polymerization rates. Even at current sequencing depths, applying our method to assess the results of a larger set of pharmacological and genetic manipulations of pre-mRNA processing factors will likely reveal new mechanisms of pre-mRNA processing and clarify interconnections between the stages of the mRNA lifecycle.

## Materials and Methods

### Accession numbers

We performed both SOLiD and Illumina RNA sequencing, available under GEO accession number GSE48889. We also rely on previously published data from GSE21161.

### Cell culture and sample preparation

#### RNA sources

Human tissue RNA-Seq was performed on total RNA from the Ambion FirstChoice Human total RNA Survey Panel. HeLa RNA-Seq was performed on HeLa total RNA isolated from HeLa cells (sequenced on Illumina for the IsoG experiments) or purchased from Ambion (cat # AM7852, sequenced on SOLiD). Mouse cortical neuron RNA-Seq data was taken from previously published work, where E16.5 mouse cortical neurons from C57B6 mice were cultured for seven days *in vitro* before isolating RNA [49]. Our human tissue analysis is IRB exempt because RNA samples were purchased as de-identified samples.

#### HeLA cell culture, RNA extraction, and qRT-PCR

HeLa cells were obtained from ATCC and were not authenticated for this study. Hela cells were maintained in DMEM supplemented with 10% fetal bovine serum, 1% Penicillin-Streptomycin, 1% non-essential amino acids. Cells were plated to about 70% confluence the day before any drug treatments on 10 cm plates. Cells were treated overnight with 30 uM Isoginketin (IsoG, Millipore) for 18 hours. For RNA extraction, cells were washed 1x with PBS then lysed with RLT buffer. Samples were immediately processed with Qiashreddar and RNeasy kits (Qiagen) and frozen until further use. 9 ug of RNA was DNAse treated and cleaned up with RNeasy Minelute kit (Qiagen). RNA quality was assessed by Bioanalyzer (Agilent) and all samples had RINs of 9.0 or higher.

#### Isolation of newly synthesized transcripts using 4SU labeling

Newly synthesized transcripts were isolated using modified methods previously described [32], [50]–[52]. Briefly, lymphoblastoid cells were pulse-labeled with 200 µM of 4-thiouridine (4SU) for 5 minutes. Cells labeled in DMSO (without 4SU) served as negative controls. Following incubation in 4SU, cells were harvested by centrifugation and RNA extracted using Trizol (Invitrogen). Extracted RNA was further purified using RNeasy columns (Qiagen) and eluted to a concentration >0.4 ng/µl. Purified RNA was denatured, and the 4SU-incorporated sites were biotinylated using 1 mg/ml EZ-link biotin-HPDP [52] by incubating at 65°C for 1.5 hours and then 25°C for an additional 1.5 hours. Unincorporated biotin-HPDP was removed twice using chloroform-isoamyl alcohol (24:1) and centrifuging the mixture in phase-lock-gel tubes (Eppendorf) as described [51]. Biotinylated RNA was captured and purified using MyOne streptavidin C1 beads (Invitrogen) and eluted in 5% β-mercaptoethanol as described [52]. RNA-seq libraries were constructed using TruSeq library preparations (Illumina).

### Sequencing library preparation

#### Illumina library construction and sequencing

RNA-seq libraries were constructed using the strand specific dUTP method [18], with minor modifications. Briefly, 3 ug of DNAse treated RNA was depleted of rRNA using Ribozero (Epicentre). Two batches of rRNA-depleted samples were combined, cleaned by RiboMinus concentration module (Invitrogen) and fragmented at 90°C for 3 min (NEB fragmentation buffer). First strand synthesis was followed by cleanup with RNAClean XP SPRI beads (Agencourt). Second strand synthesis incorporated dUTP, followed by sample clean up with MinElute PCR purification Kit (Qiagen). Fragment ends were repaired, adenylated, then ligated to True-Seq barcoded adaptors and cleaned up with AMPure XP SPRI beads (Agencourt). The libraries were then amplified by PCR for 12 cycles and cleaned up with AMPure XP SPRI beads. Illumina sequencing (1×50 bp read length) was performed on a HiSeq 2000. 4SU libraries were prepared non-strand-specifically using standard Illumina RNA-Seq.

#### SOLiD RNA-Seq library construction and sequencing

Total RNA was depleted of ribosomal RNA by hybridization using RiboMinus (Invitrogen) and was heat-fragmented, end-repaired with T4 PNK, and processed into SOLiD sequencing libraries using the double-stranded RNA ligation method in the Small RNA Expression Kit. Sequencing was performed on SOLiD with 35 bp (human tissues, mouse neurons) or 50 bp (HeLa) read lengths.

### Sequencing methods and sample list

Sequencing was performed on SOLiD using SOLiD V2 chemistry with 35 bp (human tissues) or 50 bp (HeLa) read lengths. The number of aligned reads is indicated in Table S3 in File S1.

### Summary of SnapShot-Seq model of mRNA processing times

#### Fitting the model relating mRNA processing times to densities of RNA-Seq reads

With 5 unknowns and 6 equations, our model does not have full rank. But in solving the model for individual genes, noise in the measurements leads to nonsensical solutions — i.e., negative values for timescales — for many of the genes. Based on results from simulations, we developed two methods that reliably give timescales even with noisy experimental data. In one method, we add noise to empirical read densities and slopes used to fit the model, under the assumption that our data have noise and their “true” values lie within the noise of our experimental value. We repeatedly solve the model, each time adding random noise based on experimental error to the data. We keep only those solutions having all positive timescales; upon many trials, the resulting distributions for the four processing times acquire well-defined ranges. This approach does not work for every individual intron or gene, so we employed for all figures shown a second, Monte Carlo method, whereby we aggregated the observed slopes and feature densities from random subsets of about 4,000 genes that were appropriately pre-filtered (for expression level and > 5 kb length). Samples of five genes were picked at random and, based on their aggregated slopes and densities, timescales were calculated by solving the model. This was repeated for each tissue or cell type until at least 2,000 solutions with all-positive timescales were found.

### Bioinformatics pipeline overview

#### Sequence alignment

SOLiD reads were aligned in colorspace using Corona (formerly Applied Biosystems, now Life Technologies), allowing for 0–3 or 0–5 colorspace mismatches (35 bp and 50 bp respectively). Illumina reads were aligned using BWA. In each case reads were aligned to the human or mouse genomes, plus a library of species-specific splice junctions constructed from all possible splices among annotated exons.

#### Overview of bioinformatics pipeline

After sequence alignment, RNA-Seq reads were processed using custom perl, MATLAB, and R scripts. For all analysis of RNA-Seq data by genic features, RNA-Seq data was processed by MAPtoFeatures, described below.

### Assigning RNA-Seq reads to genic features using MAPtoFeatures

MAPtoFeatures is a collection of custom-built perl scripts for assigning RNA-Seq reads to genomic features based on a given annotation. There are a number of scripts used for this work that are available upon email request. The approach is gene-centered, so alternative transcripts are merged into a representative “Gene” whose features include exons (coding and noncoding), introns, and their junctions (both before and after splicing). Reads are of fixed length (e.g., 35 bp, 50 bp) and strand-specific. The principal input files include (1) one or more Reads files, (2) a Features file, and, if available, (3) a splice library key file and (4) mappability-index files. The Reads file contains reads that have been mapped uniquely in a strand-specific manner to a genomic locus (chromosome and coordinate) or splice library. For this study, features are based on NCBI's RefSeq for mouse 37.1 or human 37.1 with exonic coordinates for annotated transcripts, namely, chromosome, strand, and locus for individual UTRs and coding exons. For each gene, reads will be assigned to the following feature categories: UTR5, UTR3, CDS (and their union, EXN), and INT; their loci are given in the Features file. Additional features that span the junctions between other features are defined to capture any reads crossing specific boundaries. These junction features include exon-intron (JXN5) and intron-exon (JXN3) splice sites and all possible intragenic exon-exon splice junctions (SPL) based on the given annotation. Existence of alternative transcription — alternative exon lengths or alternate transcriptional start sites — can obscure some features. When ambiguity exists reads are mapped to exons, *e.g.,* if a read is mapped to the sequence for an intron in one splice variant and to an overlapping exon in another variant, the read will be assigned to the exon. The Splice library key file annotates a pseudochromosome comprising every potential splice variant between two or more exons in a gene, each with minimal sequence for the junction(s) to be spanned by reads. Splice variants were derived from 27,854 RefSeq transcripts in the mouse 37.1 genome, producing 2,197,375 distinct potential splices mappable with 35-bp reads; for the human 37.1 genome, 29,149 transcripts were used to produce 2,318,291 splices. Not every feature of a gene is perfectly mappable. Mappability indices, which allow one to compensate for differences in the ability to map random reads to each feature, are created by attempting to map every potential read against all features in the Features file and splice library. In total, about 85% of EXN, 75% of INT, 60% of SPL, and 80% of JXN5 and JXN3 features are mappable with 35 bp colorspace (SOLiD) reads. The read density (coverage per target base) can then be adjusted in total or on a feature-by-feature basis to correct for mappability. Features, Reads, and splice key files can then be used to calculate the number of reads overlapping each feature of a gene. To be able to directly compare between different sequence runs, which can contain different total reads or different read lengths, read densities are renormalized for a standard total 10 million reads of length 35 bp. To obtain normalized reads densities in units of rpkm, divide our density values by 0.35.

### Determining the slope in intron density, and filtering methods

To determine the slope in intronic density, each intron was divided into 100 bins of equal sequence length, and the reads were distributed into these bins based on sequence alignment. Linear least-squared regression was used to calculate the slope of this line. Segmenting an intron into bins does not affect the calculation of slopes as compared to directly calculating a slope from the read density across a full intron. It also allows us to assess if there are aberrant regions of an intron. Accordingly, introns with spikes (often from non-coding RNA) were removed; introns whose slopes have high error were also removed.

### Read depth, counting noise, and accuracy of our model

As noted in the manuscript, our method is not applicable at single introns. We wished to determine whether this limitation was due to counting noise or technical biases. Furthermore we wished to understand the increase in read depth necessary to allow our model to be used at single intron resolution if other biases were eliminated. To calculate the expected error in our model we used Monte Carlo simulations of introns of varying lengths, with different RNA processing time scales, and different read depths. At our current average read depth, we should be able to detect intron densities, exon densities, and introns slope with 25% error for introns longer than 1 kb and a 1 minute intron degradation time. This error goes up to 160% if the intron processing time goes to 10 minutes, and drops to 8% error at intron processing times of 6 seconds. Increasing the intron to 10 kb drops the error to 7% while an intron of 100 bp increases the error to 80%. As our experimental error is significantly larger than these estimates, bias is a major limitation in our method and highlights the need for improvements in library preparation and sequence methodologies. For splice site quantification, while biases probably also exist, measurement of 5′ and 3′ splice sites are limited by counting noise. In order to achieve single intron resolution rates for the first and second step of splicing at 25% accuracy we would need approximately 50-fold more sequencing depth.

### Assessing the potential impact of alternative splicing on the calculation of timescales

Our model (Fig. 1 and Eq. 21) assumes that each intron is spliced independently, with splicing being possible as soon as all the needed intron features (5′SS and 3′SS) are transcribed. This situation is complicated by the fact that there are multiple mechanisms of alternative splicing which break this assumption. We sought to understand the potential impact of alternative splicing and other kinds of mRNA isoform diversity on our ability to calculate the timescales for each of the steps of mRNA processing. We thus assessed the frequency and potential impact of a variety of alternative isoforms, taken from the literature [20]:

#### 1. Skipped exons

We cannot accurately filter skipped exons out of our analysis, but according to our analysis of exon junction RNA-Seq reads, only ∼2% of exon junctions detected in total RNA correspond to skipped exons (Fig. S1F), limiting the effect of skipped exons on our lifetimes to 2%. Skipped exons will increase the 5′SS, 3′SS, and intron density and thereby the apparent lifetime of each feature by the same amount – namely the time required to transcribed the skipped exon and the following intron. The exonic density and splice junctions will decrease. While, in theory, T_5_ is included in equation 21e, in practice T_μ_ dominates this equation, as it is the longest times scale. Hence, the change cause by skipped exons can be lumped total into the T_5_ – this is the term shared between equations 2–4. This makes intuitive sense in that a delay in splice will also add the same amount of density to the 5′ SS, 3′SS and intron. Importantly, this means that our calculation for T_5_ is an upper bound on the rate. The decrease in T_μ_ should be minimal as only a small portion of a gene's exonic density comes from any one exon.

#### 2. Alternative 5′ or 3′ SS

There are two scenarios: one where the alternative splice-site shortens the intron, and one where it lengths in the intron. When the intron is lengthened, the exon density will decrease slightly (because it is degraded with the intron) and the 5′ or 3′SS will increase slightly as they will now live as long as the intron. 5′ alternative splice sites will result in a slight over-estimate of synthesis rates and an underestimate of T_3_ and T_γ_. 3′ alternative splice sites will result in T_3_ being over estimated and T_γ_ being underestimated. If the intron is shorter than expected the 5′SS and 3′SS will become part of the intron leading to significantly higher density and the potentially to greatly overestimate the T_5_ and T_3_. Luckily, shorter introns can be filtered out as it causes the 5′SS and 3′SS density to increase from a level that is similar to the intronic read density to a level that is similar to the exonic read density.

#### 3. Unannotated alternative first/last exon

These events should have no effect on our model because we treat each intron or splice unit independently.

**4. Unannotated tandem 3′ UTRs** won't affect our model, since they are found outside the genic regions we consider.

**5. Mutually exclusive exons** are a special case of skipped exons and will have them same effects on rate calculations as skipped exons.

**6. Retained introns** are relatively rare [20]and would behave similarly to skipped exons except that the increase in signal is now large as it depends on the lifetime of the mRNA as opposed to the time required for alternative splicing. This could lead to a major over-estimate of T_5_, but again, we can filter out retained introns as it causes the 5′SS and 3′SS density to increase from a level that is similar to the intronic read density to a level that is similar to the exonic read density.

In summary, most alternative splicing can be either ignored, as it doesn't affect our model (e.g. unannotated first exons), or can be filtered out because of its observable effect on read density (e.g. retained introns). The remaining alternative splicing events (*e.g.,* skipped exons) affect our model but do this by causing us to overestimate T_5_ or slightly underestimate T_μ_. As T_5_ is already relatively low, the fact that alternative splicing serves as an upper-bound gives increased confidence that the true value is at most the value we calculate. While our T_μ_ could be an underestimate, the amount of decrease in this rate should be proportional to the frequency of skipped exons which is a relative small portion of total exonic counts (Fig. S1F) [20].

### The SnapShot-Seq model relating mRNA processing times to densities of RNA-Seq reads across genic features

Our model of the eukaryotic mRNA life cycle aims to extract dynamical information about RNA transcription, processing and degradation from a single pool of RNA-Seq reads. The organizing assumption of this model — that the biological sample, or samples, from which the reads are derived has attained a steady state with respect to these processes — allows us to assert that all species of nascent, premature, or mature RNA and their derivatives are present in the RNA-Seq library in direct proportion to their mean lifetimes in the cells from which they were obtained. Thus, if we know relative abundances of the different RNA species that arise in our model, we should conversely be able to infer RNA-processing lifetimes from experimentally determined feature abundances. Equivalently, specific genomic features such as introns, exons, and their junctions acquire different relative abundances in the various stages of RNA processing and so allow us to predict lifetimes from genomic locus-specific RNA-Seq data.

Our model is based on the following assumptions about the structure, creation, splicing, and degradation of mRNA transcripts:

Each gene comprises *N*+1 exons separated by *N* introns (*N*≥0), whose lengths are all arbitrary, unless otherwise indicated.
Transcription of each gene *g* is initiated randomly at a fixed rate of *S_g_* transcripts per second. This rate is independent of how long the polymerase complex might have been “loaded” at the transcriptional start site. Once begun at the 5′-most exon, transcription proceeds uniformly and continuously through all features at an average, transcript-independent rate α =  3.6 kb/min, i.e., with a characteristic time constant *T*_α_ = 1/α = 1 sec per 60 bp. If transcriptional pausing occurs, we assume that it arises randomly throughout the transcript and effectively lowers the value of α; pausing that preferentially occurs at specific features, e.g., at the beginning of exons, can in principle be identified as a consistent kink in the differential slope across those features. Transcription terminates when the polymerase reaches the 3′ end of the 3′-most exon — we ignore details concerning the transcript's 5′ cap and polyA tail.
Splicing out of each intron takes place in two steps, each with its own characteristic time. (1) Lariat formation occurs at a time *T*_5_ after the polymerase has transcribed the 3′ end of the intron; at that time the 5′ splice site (5′SS) between the intron and its upstream exon is cleaved and hence the junction feature there is destroyed. (2) Exon-exon splicing is completed at a time *T*_3_ after lariat formation, whereby the 3′ splice site (3′SS) between the intron and its downstream exon is cleaved, that junction feature is destroyed, and the adjacent exons are ligated together. We assume for now that these two events simply cut the first and last bases in the intron from their neighboring exonic bases but that the entire intron remains accessible to RNA-Seq. This standard model of splicing assumes that transcription proceeds apace, without locus-specific pausing, even during the time *T*_5_+*T*_3_ these splicing steps are carried out. As a result, each intron is spliced out in sequence but independently. Once an intron has been removed, it is assumed to persist in the sample for a further length of time *T*_γ_, at which point it is degraded and no longer detectable. We ignore alternative splicing here; our data suggest that, although some exons may be occasionally skipped in most genes, only 2% of all splicing events involve nonadjacent exons (not shown).
The lifetime of a mature mRNA transcript is taken to begin as soon as the 3′-most pair of exons have been ligated following intron excision. The mRNA is assumed to exist, and all of its constituent exons to be detectable, for a time *T*_μ_, after which it is considered degraded and undetectable.
Excised lariat degradation does not contribute to the intron expression profile. Although this assumption is consistent with the observations in Fig. S1, the precise mechanism of excised lariat degradation is unknown, and these observations do not rule out every possible contribution of lariat degradation to the intron expression profile.

From this model we can predict the duration of every single transcribed base, from the primary transcript's synthesis until the introns' and mature transcript's degradation. The abundances of bases at individual loci or throughout genomic regions are quantified here by their relative coverage by RNA-Seq reads of length *r*. The times {*T*_5_, *T*_3_, *T*_γ_, *T*_μ_} and synthesis rates *S_g_* are to be determined.

For purposes of illustration, we begin by assuming that a transcript has *N*+1≥2 exons of equal length Λ and *N*≥1 intervening introns of equal length *L*>>1. Consider first the fate of any one of the introns. After transcription brings into existence the RNA bases at its 5′ end, those bases will continue to exist as part of nascent transcripts while the remainder of that intron continues to be transcribed, for some duration *T*_t_. They further persist throughout intron processing — during the two stages of splicing and until the whole intron has been degraded, i.e., with an additional duration equal to the total processing time *T*_p_ = *T*_5_+*T*_3_+*T*_γ_. Bases near the 3′ end, on the other hand, are created at the termination of the transcription “clock” and so endure only for the time *T*_p_ following transcription. In general, a base located at a distance *x* from the intron's 5′ end (0≤*x*<*L*) has a “transcription waiting time” *T*_t,INT_(*x*)  =  *T*_α_(*L–x*) during which it is part of some nascent transcript in which that intron has not yet been completely transcribed. The total duration of such an intronic base is 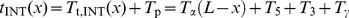
(1)


The abundance of this base in a real sample will be proportional to this total time — derived for a single transcript — times the rate *S_g_* at which transcripts were initiated in the sample. The number of such bases therefore equals *t*_INT_(*x*)·*S_g_*. We quantify feature abundances from RNA-Seq data with single-base resolution in terms of reads “Density” *D* (see the Methods section on MAPtoFeatures). Since this quantity depends on the concentration of total RNA in the sample, depth of RNA sequencing, and normalization of total reads number, we must introduce a sample-specific proportionality constant *c*_0_ (units Density per transcript base counted) to rescale the relative abundances of all features measured in the same sample to Densities: 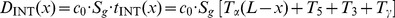
(2)


The Density of reads over the entire intron follows from averaging Eq. (2) over all *x*: 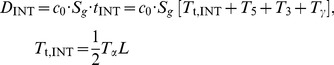
(3)


where *t*_INT_ is the average duration of intronic bases. Note that *T*_t,INT_, the average transcription waiting time for all bases in the intron, employs half its length. This result applies to every individual intron of length *L*. Moreover, the average Density over all intronic bases in the gene is trivially the same in this case, because all introns have the same length. The *change* in Density per unit length across each intron is given by the slope 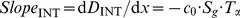
(4)which offers a direct readout of a gene's relative mRNA synthesis rate. (As a rule of thumb, for each gene *g*, the value 3600·|*Slope_g_*| gives *c*_0_ *S_g_* in units of normalized Density accumulated per minute of feature duration.) In this model, the Density in an intron always falls from its 5′ to its 3′ end, at a constant rate.

Junctions between exons and introns, the 5′SS and 3′SS features, behave similarly to the introns' ends except they do not last until intron degradation. Each intron's 5′ splice site is created as soon as transcription of the intron begins, waits a time *T*_α_ *L* for the entire intron's transcription, and then disappears at the first splicing step, after time *T*_5_, so its total duration is *t*_5′SS_  =  *T*_α_ *L*+*T*_5_. Its 3′ splice site is instead created after the intron's transcription and is destroyed after *t*_3′SS_ *=  T*_5_+*T*_3_, the time it takes for both splicing steps. The associated Densities are 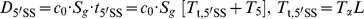
(5)

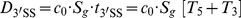
(6)


These expressions relate the abundance of reads that cross either splice site to the lifetimes of these junctions. If all splice-site features in the genome can be regarded as having equal length σ = *r*–1, then Eqs. (5) and (6) also characterize the average Densities of reads crossing all splice-site junctions of either kind in a gene. It is also useful to compare the change in Density between intron ends, 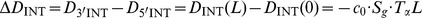
(7)to the difference between the Densities at a pair of splice sites: 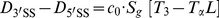
(8)


The former extrapolates to zero as *L* → 0 whereas the latter equals zero when *L* = *T*_3_/*T*_α_, which is exploited to directly infer processing times from Densities binned by intron length.

The durations and Densities of exons and splices are only slightly more complicated. Consider the first exon. Precisely as described above for introns, the base at position *x*_1_ in this exon (0≤*x*_1_<Λ) has a transcription waiting time *T*_α_(Λ*–x*_1_) for the rest of that exon. Unlike introns, however, the next relevant step is not intron processing but rather more transcription. In particular, every base in the first exon further endures for the whole time it takes to transcribe the *N* downstream introns and exons. If we count all but the 3′-most exon, the total transcription waiting time for this base, up to the end of the last intron, equals 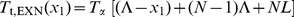
(9)


The countdown to degradation of the mature transcript after a time *T*_μ_ begins once the splicing of the final exon pair has been completed. This depends on the longer of two processes: the total time *T*_5_+*T*_3_ to splice out the final intron *vs.* the time *T*_α_Λ for transcription of the final exon. The median length of 3′-most exons in mouse and human (which are generally long 3′UTRs) are 905 bp and 1036 bp, respectively; their exon transcription times are typically less than 20 seconds, whereas we find that in all tissues the mean total splicing time is over 3 minutes. Thus, after the last intron, it will be usually correct to assume that there is an additional waiting time *T*_5_+*T*_3_, followed by the (much longer) lifetime *T*_μ_ of the mature mRNA proper until its degradation. Generalizing to exon number *e* (1≤*e*≤*N*), the base at position *x_e_* in that exon has total duration 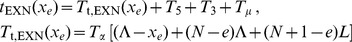
(10)


To be consistent, durations for bases in the very last exon (*e* = *N*+1) have to be treated as a special case. The 5′-most base of this exon is transcribed just after transcription of the last intron has completed, so this base waits the full time *T*_5_+*T*_3_ for splicing of that intron to finish. However, bases lying farther downstream of the intron, at exonic position *x_N_*_+1_ >0, are created later and thus have durations that are *shorter* than that of the 5′-most base by an amount equal to the lag in transcription start time for the base at *x_N_*_+1_. Hence bases in the final exon have total duration
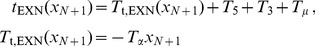
(11)


[Of course no duration here is actually negative; this exon's bases have lifetimes equal to the *total* given in Eq. (11).] Since Eq. (10) with *e* = *N*+1 yields the same results as Eq. (11), Eq. (10) can now be applied to all exons (1≤*e*≤*N*+1). The Density at base *x_e_* of any exon *e* is therefore 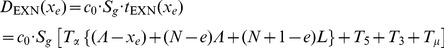
(12)


The average Density over an entire exon follows from averaging Eq. (12) over all *x_e_*: 
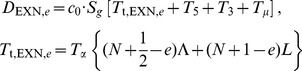
(13)


For the average Density over all exons in a gene, we turn to our definition of Density as total rdbp, the total count of all bases of all reads of length *r* that overlap exons, divided by bp, the total number of bases available in exons (see the MAPtoFeatures section). For exon number *e*, with length bp*_e_*  = Λ, we have rdbp*_e_*  =  *D*_EXN,e_ · bp*_e_*, so the ratio of their totals (sums over *e* = 1, …,*N*+1) gives the average exonic Density 
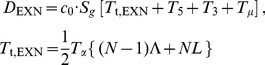
(14)


Unlike Eqs. (3) and (5) for introns and 5′ splice sites, for which the transcription waiting time involves a single intron, Eq. (14) shows how newly transcribed exons persist while all downstream exons and introns (half, on the average) are also transcribed.

Finally, exon-exon splice junctions come into being as soon as the second step of joining splice sites is complete; these junctions survive as part of the mature transcript until it is degraded. The duration of splice number *s* (1≤*s*≤*N*), from transcription of the next exon up through the last intron, equals the total transcription waiting time of *N*–*s* exons and *N*–*s* introns, less the initial time *T*_5_+*T*_3_ for this splice to actually have been finished. Its remaining time, as for the last exon, equals the waiting time *T*_5_+*T*_3_ for the last splice to finish (which cancels out in the total) plus the subsequent time until mature-RNA degradation. In total, the Density of a single exon-junction feature is 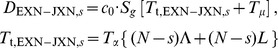
(15)


For the average Density over all splices in a gene, we assume for the moment that each splice has the same effective length bp*_s_*  = *r*–1; then the ratio of total rdbp*_s_*  =  *D*_EXN-JXN,*s*_·bp*_s_* and total bp*_s_* (sums over *s* = 1, …,*N*) yields 
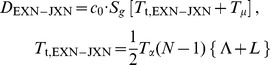
(16)


As for the average exon Density, Eqs. (13) – (14), the average exon-junction Density includes an average waiting time for transcription of downstream introns and exons.

This simple version of our model for a generic transcript is summarized by Eqs. (5), (6), (3), (14), and (16), which express relations between measurable Densities for the five features {5′SS, 3′SS, INT, EXN, EXN-JXN} and the unknown synthesis rate *S_g_* and four processing times {*T*_5_, *T*_3_, *T*_γ_, *T*_μ_} (and the constants *c*_0_ and *T*_α_). Importantly, a sixth relationship, Eq. (4) or (7), also expresses *S_g_* directly as a change in Density across single introns. These six expressions are yet further reduced for presentation in Fig. 1, where we make the additional assumption that exon length is negligible compared to intron length (Λ<<*L*) and call the single-intron transcription time *T*_t_  =  *T*_α_ *L*. In all applications of the model, however, all Densities for a gene remain proportional to the factor *c*_0_ *S_g_*.

The general version of our model lifts the artificial equal-length restriction on exons and introns. For individual features, this only affects the expressions involving transcription waiting times for exons and exon-exon splice junctions, Eqs. (12) – (13) and (15), which depend on multiple downstream feature lengths. [Intron number *i* and its 5′ splice-site junction depend on a single intron length *L* = *L_i_* in Eqs. (3) and (5).] Allowing any exon lengths {Λ*_e_*, *e* = 1, …,*N*+1}, intron lengths {*L_i_*, *i* = 1, …,*N*}, and effective splice-feature lengths {*λ_s_*≤*r*–1, *s* = 1, …,*N*}, we find 
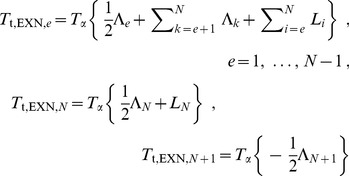
(17)[where again it is understood that the negative time for *e* = *N*+1 will be added to *T*_5_+*T*_3_+*T*_μ_, as in Eq. (11)] and 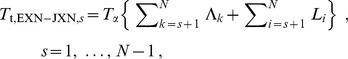
(18)with *T*_t,EXN-JXN,*N*_ = 0. To calculate the average Density over all features of a certain type in a gene, we again note that the total contribution of reads to a feature's bases equals rdbp*_i_*  =  *D_i_*·bp*_i_* for feature number *i* with density *D_i_* and length bp*_i_*; from these we find the average Density by dividing the reads' total contributions to all feature bases by the total number of bases available for mapping: 
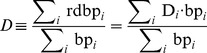
(19)


Thus, from the individual introns' Densities in Eq. (3) we obtain (with bp*_i_*  =  *L_i_*) 
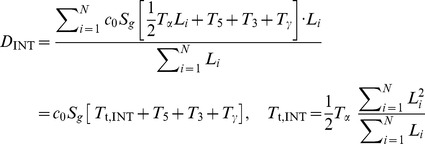
(20)


Squared lengths appear here and for other features because both the average transcription waiting time per base and the number of bases in the feature are proportional to feature lengths. Also note that the constants *T*_5_, *T*_3_, *T*_γ_, and *T*_μ_ common to all features will appear linearly; details of feature lengths enter only into *T*_t,INT_ and the other mean transcription waiting times.

Overall Densities for the other types of features are calculated in the same way, namely, as the length-weighted average of their individual features' Densities. We collect all the results here in a set of equations that defines the most general version of our model of the mRNA life cycle: 
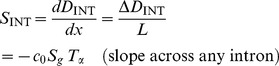
(21a)

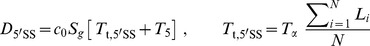
(21b)

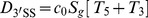
(21c)

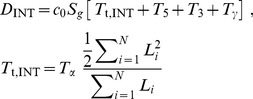
(21d)

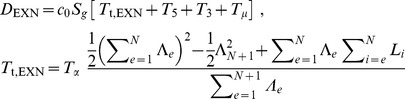
(21e)

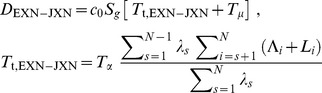
(21f)


These six equations generalize those displayed in Fig. 1. This form of the model applies to genes with one or more introns (*N*≥1). A singleton exon (*N* = 0) would have *D*_EXN_  =  *c*_0_·*S_g_* [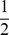 *T*_α_Λ+*T*_μ_] and zero Density for the other features; for *N* = 1, there is one splice but *T*_t,EXN-JXN_  = 0. The dependence of transcription waiting times on feature lengths tends to be messy; though their values might be dominated by long introns, the longest introns are usually the first two, which also happen to appear least often in the sums in Eqs. (21e–f), so these expressions do not generally simplify.

In principle, for each gene *g*, the solution of Eqs. (21a) – (21f) for the 5 unknowns {*S_g_*, *T*_5_, *T*_3_, *T*_γ_, *T*_μ_} depends straightforwardly on: the precise measurement of expression levels {*D*} for 5 different kinds of genomic features, the calculation of transcription waiting times {*T*_t_} from known feature lengths, and acquisition of experimental values for the transcription time *T*_α_ and sample concentration constant *c*_0_. In practice, there are several complications.

Firstly, this set is overspecified — there are 6 equations for 5 unknowns. If we were to ignore Eq. (21a), the remaining 5 equations could be divided by *S_g_* to yield 5 equations linear in 5 unknowns: the inititation time 1/*S_g_* and the four processing times, which we abbreviate {*T*}  =  {*T*_5_, *T*_3_, *T*_γ_, *T*_μ_}. These equations are, however, all homogeneous in these 5 variables, in which case at most 4 of the variables are independent, leaving us once more with an overspecified set of equations. Equivalently, we could substitute the value for *c*_0_ *S_g_* obtained directly from Eq. (21a) into Eqs. (21b–f), which would yield 5 inhomogeneous equations in the 4 unknowns {*T*}. One could solve such sets of equations using linear least-squares methods or some other optimization procedure to obtain “best” values of {*T*} for individual genes, including errors of these estimates, along with the measured synthesis rates *S_g_*.

Furthermore, two features of the data conspire to defeat this approach: (1) the Density data for single genes are fairly noisy, most especially the slopes *Slope*_INT_, and (2) the “degree of independence” between Eqs. (21e) and (21f), for exons and exon-exon junctions, is minimal. The latter point hinges on the fact that mature-transcript degradation times *T*_μ_ typically turn out to be an hour or more — always much greater than the transcription times *T*_t_ or processing times {*T*_5_, *T*_3_, *T*_γ_}, which are usually just a few minutes. The righthand sides of the equations for *D*_EXN_ and *D*_EXN-INT_ therefore tend to be nearly equal, relegating these expressions to a consistency check rather than independent information towards a solution. Noisiness in the Density data is a by-product of the RNA-Seq technique and biological variability, but to the extent that it is due to counting noise this should be less of an issue at greater sequencing depth. Also, note that each rate *S_g_* cannot be determined in absolute units of transcripts initiated per second — only in the combination *c*_0_ *S_g_* (Density per time) — unless sample preparation has allowed for an estimation of *c*_0_.

The precise measurement of intron-Density slopes *Slope*_INT_, Eq. (21a), in order to obtain relative synthesis rates *c*_0_ *S_g_*, presents several obstacles. The distribution of our measured Densities across single introns always has a lot of scatter from sequencing noise, variations in mappability, and spikes from randomly inserted repetitive elements. Nevertheless, the longer the intron, the more likely a straight-line fit would yield a negative slope significantly different from what would be expected from random Densities. Such fits produce roughly log-normal distributions of negative-slope magnitudes |Δ*D*_INT_/*L*| centered around 10^−6^ –10^−5^ (i.e., *c*_0_ *S_g_* ∼0.01 min^−1^) but spread out over 3–4 orders of magnitude. Despite this wide range of values, when introns of approximately the same length are aggregated together, their median slope values are remarkably consistent between length bins; moreover, aggregated introns generally exhibit the expected negative slope as a function of distance from either 5′ or 3′ end. (Short introns have Density distributions that are so noisy, however, that positive or negative slopes mostly occur randomly — another reason for aggregation.) These results suggest that a practical approach to extracting realistic solutions from the model Eqs. (21a–f) should involve the aggregation of synthesis rates. Introns specifically from the same gene, which share the same synthesis rate, ideally should have negative slopes that reflect this common rate, taking into account their fitting errors — but even within genes our data for intron Densities have enough noise to impose excess variability on slopes.

We employed the following heuristics, first to decide whether slopes *Slope_g,i_* fit to individual introns were sufficiently informative, and then to combine intra-genic slopes to estimate a representative value of *c*_0_ *S_g_*  =  –*Slope_g,i_*/*T*_α_ for each gene *g*. As described in the MAPtoFeatures section, each intron was divided into 100 equally-spaced bins to which normalized read Densities were assigned. The slope *Slope_g,i_* of every intron *i* and the slope's standard error *ε_g,i_* (SE) were calculated from a simple linear least-squares fit to bins with nonzero Densities {*D*_INT,*g,i*_}. A goodness-of-fit parameter was taken to be the true-discovery rate (TDR) under a null model, defined here as equal to one minus the one-tailed *P*-value for obtaining this slope value or less with this error from random data; more significant negative values thus correspond to TDR*_g,i_* → 1. Within each gene, only introns at least 5 kb in length and with slopes fit to at least 20 bins were considered usable. Genes that had no such introns were not used in solving the model for {*T*}. Candidate genes were assigned a single slope *Slope_g_* equal to the arithmetic mean of the slopes {*Slope_g,i_*} of their surviving introns; a propagated error *ε_g_* for *Slope_g_* was calculated by combining these introns' slope errors {*ε_g,i_*} in quadrature (the SEs were weighted in proportion to intron lengths and squared TDRs); and a final TDR*_g_* for *Slope_g_* was calculated from a *z*-score *Slope_g_*/*ε_g_*. Further filters applied to genes used to solve the model were TDR*_g_* ≥0.90 (only the best-consensus slopes), average *D*_INT,*g*_ ≤1.00 (to remove spikes in Density), average *D*_EXN,*g*_ ≤50 (to avoid bias from a few very highly expressed genes), and coding genes only. For our data sets these steps typically left 1,500–4,000 genes {*g*} having slopes deemed adequately reliable for input into Eq. (21a).

The system of 5 equations (21b–f) for the 4 unknowns processing times {*T*_5_, *T*_3_, *T*_γ_, *T*_μ_} nevertheless tends to suffer instabilities in the following sense. Whether we try to fit the times using this overspecified set or drop Eq. (21f) and solve for the {*T*} exactly, solutions based on feature Densities for a single gene often include one or more negative times. Such solutions are not interpretable in our model and should be rejected. In order to sidestep this issue and still extract a representative range of values for {*T*} consistent with the assumptions of our model, we considered two approaches. We could adjust the various Density distributions in Eqs. (21a–f) by adding some amount of random noise, within the limits of our empirically observed noise, that would optimize the number of genes with “sensible” solutions, namely, with {*T*>0} (all times positive). After all, it is noise in our data that is presumably perturbing this system of equations out of the region of {*T*}-parameter space that contains “stable” solutions. Or we could perform Monte Carlo simulations, in which we would sample the set {*g*} of filtered genes, aggregate their observed slopes and feature Densities, and solve the model for such aggregated samples, repeating as necessary.

In order to calculate lifetimes, we adopted the second approach, leaving out Eq. (21f) for exon-exon splice junctions and solving the set of Eqs. (21b–e) for {*T*} using random samples of *N* genes as follows. (Note, however, that to account for errors in the estimated slopes we do add random gaussian noise to each gene's mean slope based on its inferred SE.) For each sample, the genes' slopes are geometrically averaged and divided by *T*_α_ to yield an aggregated value of their synthesis rate, *c*_0_ *S*  =  –Δ*D*/Δ*T*. We similarly aggregate the sample's nonzero Densities for each feature type via their geometric mean (zero Densities are ignored; any sample with only zero Densities for one or more feature types is discarded). Each sample's aggregated Densities are regarded as a vector ***D***  =  (*D*_5′SS_, *D*_3′SS_, *D*_INT_, *D*_EXN_), as are the unknown processing times ***T***  =  (*T*_5_, *T*_3_, *T*_γ_, *T*_μ_) and known transcription waiting times ***T*** **_t_**  =  (*T*_t,5′SS_, *T*_t,3′SS_ = 0, *T*_t,INT_, *T*_t,EXN_), while the coefficients of ***T*** on the righthand side of Eqs. (21b–e) comprise a 4×4 matrix ***M*** of zeros and ones. This linear system of 4 equations is solved at once by inverting ***M***: 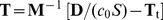
(22)


The result is considered valid only if all four processing times are positive. We gather the valid solutions for many random samples and characterize their distributions of *T*_5_, *T*_3_, *T*_γ_, and *T*_μ_, each of which tends to be consistent with a log-normal distribution (not shown). By following this procedure for our pooled data set of 140 million reads from total RNA taken from mouse cortical neurons, we found 8,517 sets with {*T*>0} out of 200,000 random samples of 5 out of 2,338 mouse genes. For each human tissue, we ran series of 200,000 random samples of size 5 until at least 2,000 valid solutions were found. Table S4 in File S1 shows the numbers of total reads, filtered genes used, Monte Carlo samples, and valid solutions per tissue.

Our chosen sample size of *N* = 5 was an empirical compromise balancing the advantage of aggregating several genes — to probe the range of each processing time with greater precision — against the rarity of useful solutions for too large a sample (e.g., *N*>20). Although widths of the calculated processing-time distributions depend on sample size, they are ultimately limited by the large variability of the inferred intronic slopes and, to a lesser extent, the spread of each kind of feature Density.

### Statistics, reagents, and animal models

#### Sample sizes and Replicate numbers

From prior sequencing runs, we had determined that the sequencing depth we used here (∼15 M–100 M reads per sample) would allow us to obtain variations in expressions levels over at least 7 orders of magnitude, which was more than sufficient for our analyses. Similarly, previous analyses had suggested that large differences between samples could be detected using 2–3 biological replicates. We sequenced only one biological replicate each for our human tissue samples, so we do not make any claims about significant differences between tissues. We sequenced three biological replicates for our isoginkgetin and control treatments. No samples were excluded from analyses. For animal experiments, no randomization was used, and no randomization was used to determine how samples/animals were allocated to experimental groups. No blinding was used.

#### Additional details regarding statistical treatment in Fig. 2

In Fig. 2, processing times for intron splicing, intron decay, and mRNA decay are obtained from Monte Carlo simulations, typically several thousands, as described in our discussions of implementing the SnapShot-Seq model; displayed error bars indicate either standard error (SE) or, based on the assumption of normality, or ∼1.96 SEs to illustrate 95% confidence intervals; sample sizes are enumerated in the table below supplemental Eq. (22).

#### Distributions of data and statistical test choice

Most of the data used to generate our figures involve distributions of RNA-Seq reads from individual samples whose quantification is mostly a matter of binning and counting. Explicit mention is made when technical or biological replicates are pooled. Distributions of expression levels and most other quantities presented here tend to be log-normal, though upon sufficiently deep sequencing Density distributions exhibit an additional low-expression mode discussed in the text. Other distributions displaying principally log-normal behavior include: expression fold changes and other ratios, such as mRNA stability (D_EXN_/D_3′INT_); distances between genes and between intragenic features; feature lengths; intronic transcription times (which are proportional to intron length); and RNA processing and decay times. We therefore use parametric unpaired *t*-tests to compare samples between like distributions, unless otherwise indicated. Note that read counts within individual features are proportional to Densities as well as feature lengths and hence do not obey simple Poissonian statistics. Variation within data groups was similar unless otherwise noted.

#### Animal models

All animal experiments were approved by the Harvard Medical School IACUC committee.

## Supporting Information

Figure S1 The decrease in expression across introns is not affected by excised lariat degradation or alternative splicing. (**A–D**) Both transcription and intron degradation could in theory contribute to the slope of intronic expression. (**A**) Five timelines of the intron lifecycle, each with different assumptions about the mechanisms and rates of intron degradation. Models I and IIa assume a 3′-to-5′ intronic exonuclease with a rate either equal to (I) or 10-fold faster (IIa) than that of RNA polymerase II (RNAPII). Model IIb assumes such a rapid exonuclease rate (>> 10× that of RNAPII) that it contributes negligibly to the intronic expression profile. Models IIc and III assume 5′-to-3′ exonucleases with rates ten times faster than (IIc) or equal to (III) that of RNAPII. (**B**) Models I, IIb, and III from **A**, with 5′ and 3′ splice sites (SSs) shown, emphasizing that intron degradation does not affect splice site abundances. (**C**) For models I, IIb, and III from **A**, the resulting intronic and splice site expression profiles. (**D**) For all models, predicted changes in the difference in expression between 5′ to 3′ ends of introns (or SSs) with increasing intron length. Only when the rate of exonucleolytic intron degradation is much faster than the rate of RNAPII are the slopes for intron ends and splice sites equal. (**E**) The decrease in intron density across introns and the decrease in density between 5′ and 3′ SSs are similar over a wide range of intron lengths, ruling out a significant contribution of excised lariat degradation to the intronic expression profile. Error bars represent s.e.m. from multiple introns from a single representative biological sample. (**F**) Approximately 1% of exon-exon splice events are between non-consecutive exons, while ∼3% of annotated exons are detectably non-consecutively spliced. We required non-consecutive events to be detected by at least two sequencing reads. Error bars are s.e.m. from 3 mouse cortical and 5 HeLa biological replicates. All data are from mouse neurons (using SOLiD), except where noted. HeLa data were generated using the dUTP/Illumina method.
(PDF)

Figure S2 Increases in intron relative to exon expression in isoginkgetin-treated cells are not limited to a few genes, and how to interpret the sawtooth pattern of intronic expression across genes. (**A**) Most or all genes have increased intron to exon expression levels upon isoginkgetin treatment. The *x*-axis shows a ratio of ratios, indicating the increase in the intron to exon ratio upon isoginkgetin (IsoG) treatment. (**B**) Co-transcriptional versus obligate post-transcriptional models of splicing can be distinguished by how intron read density changes across a gene. In a model of co-transcriptional splicing in which splicing can occur at any point after the 3′SS has been transcribed (blue), the density at the 3′ end of each intron in a gene is predicted not to vary systematically along a gene. In obligate post-transcriptional splicing, in which splicing can only occur once transcription of the entire gene is complete (orange), intron density declines continuously from one intron to the next from 5′ to 3′. Decreases in intronic expression due to a uniform rate of premature termination would also be predicted to match the orange model. Regardless of the rate of splicing, the blue model holds as long as splicing is able to occur upon completion of intron transcription.
(PDF)

Figure S3 mRNA synthesis rate can be inferred from the density of reads across introns. The slope of intron density (ΔD_INT_/L_INT_) is proportional to the mRNA synthesis rate (Fig. 1A) but is difficult to measure precisely due to counting noise. Intron density (D_3′INT_) may be measured with greater precision, but it is proportional not only to the mRNA synthesis rate but also to the time of intron processing (*T*_p_ = *T*_5_ + *T*_3_ + *T*γ). Nonetheless, if the variability in intron processing rates were low enough relative to the variability in synthesis rates, D_3′INT_ would be a useful proxy for synthesis rate. (**A**) To evaluate the utility of intron density (D_3′INT_) as a proxy for synthesis rate, we plotted ΔD_INT_/L_INT_ versus D_3′INT_ (total RNA-Seq, mouse neurons) and found a linear least squares log-log slope of ∼1, indicating nearly direct proportionality between ΔD_INT_/L_INT_ and D_3′INT_. (**B**) The relationship shown in panel **A** holds across ten human tissues. (**C**) 4SU-Seq exon densities and total RNA-Seq *exon* densities are not linearly related (*i.e.,* do not have a fit slope of 1 on a log-log scale). This result is in contrast to that for 4SU-Seq exon densities and total RNA-Seq 3′ *intron* densities (Fig. 4D). To make this distinction more obvious, panel (**D**) shows an overlay of scatterplots from Fig. 4D and panel (C). To confirm that RNA-Seq 3′ intron densities are a better indicator of mRNA synthesis rates than RNA-Seq exon densities across the full range of expression levels, we computed fits for low expressors vs high expressors (subsets of the data in panel D). Fit slopes were 0.88 (low expressors) and 0.84 (high expressors) for 4SU densities vs. RNA-Seq 3′ intron densities, and fit slopes were 0.90 (low expressors) and 0.72 (high expressors) for 4SU densities vs. RNA-Seq exon densities. We speculate that expression levels correlate more tightly with synthesis rates within the low mode of gene expression due to the ∼5-fold lower (median) mRNA stabilities in this mode (see panels E–F). (**E–F**) Within each mode, synthesis rate and mRNA stability contribute equally to expression levels, as indicated by the fact that the width of each distribution is similar (FWHM, Full-Width-Half-Max). While both synthesis rates and mRNA stability are decreased from the high to the low mode, synthesis rate decreases more. The high mode is shown in **E**, and the low mode is shown in **F**. RNA-Seq data is from lymphocyte cell lines in C–D and from mouse neurons in E-F.
(PDF)

Figure S4 Bimodality of gene expression is driven by bimodality of mRNA synthesis. (**A**) Distributions of gene expression for each of ten human tissues sequenced using strand-specific total RNA-Seq on SOLiD. (**B**) Genes in the low or high modes of gene expression are also respectively in the low or high modes of mRNA synthesis, *i.e.,* the low end of the *x*-distribution is also the low end of the *y*-distribution (data from mouse neurons). (**C–E**) As a further test of the bimodality of pre-mRNA synthesis rates but not mRNA stabilities, we confirmed that our 4SU exon (C) and intron (D) densities were bimodal. In contrast, mRNA stability was unimodal (E), even when computed using 4SU data. In (E), mRNA stability was estimated from total RNA-Seq exon densities (representing expression) divided by 4SU exon densities (representing synthesis) from the same lymphocyte cell line.
(PDF)

File S1 Contains Tables S1, S3, and S4. **Table S1.** Average mRNA lifetimes by Tissue. **Table S3.** Counts of uniquely aligned reads for each RNA-Seq library sequenced. **Table S4**. Summary of model solutions by tissue.
(XLSX)

Table S2 Gene Ontology analysis, using DAVID, of high expressors, low expressors, and genes with no detectable expression.
(XLS)
