# Supplementary figures and images for: SnapShot-Seq: A Method for Extracting Genome-Wide, In Vivo mRNA Dynamics from a Single Total RNA Sample
Source: PLoS One. 2014 Feb 26;9(2):e89673. doi: 10.1371/journal.pone.0089673 (PMC3935918; doi:10.1371/journal.pone.0089673)

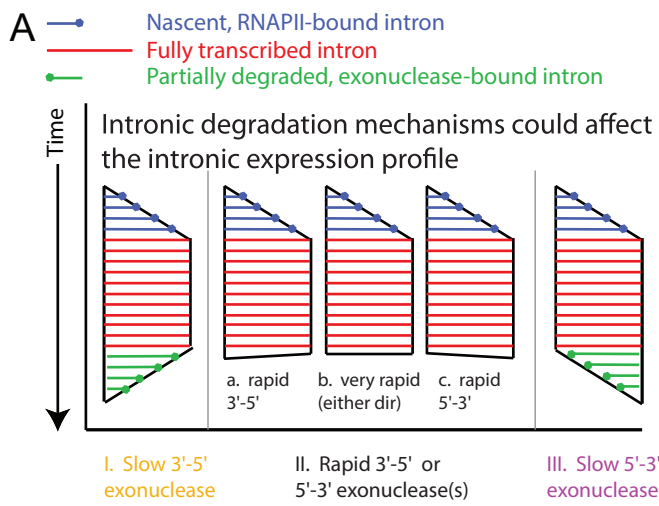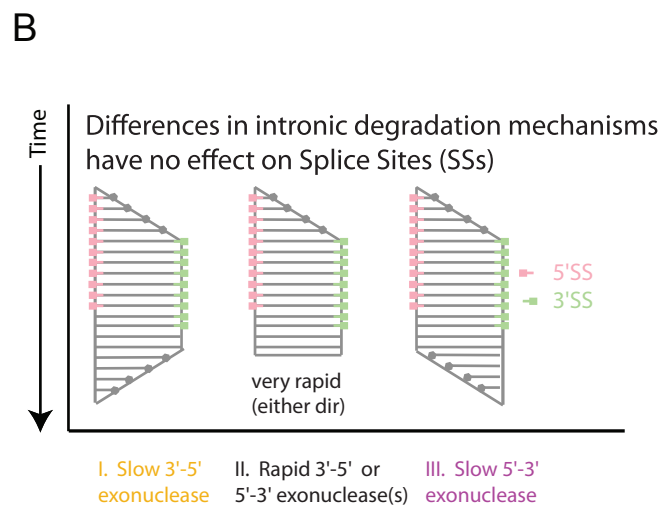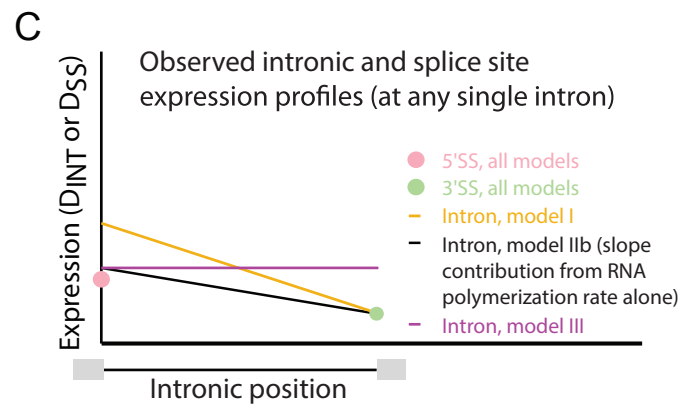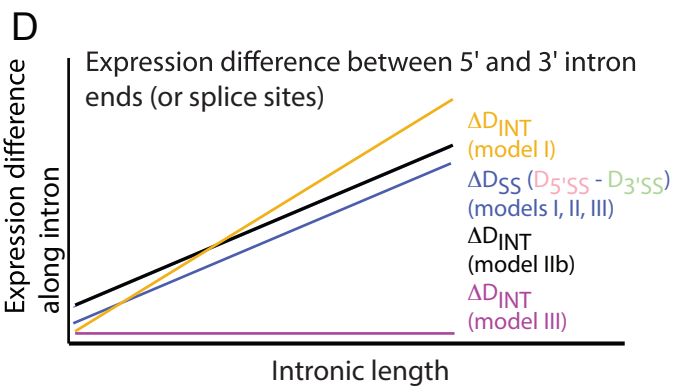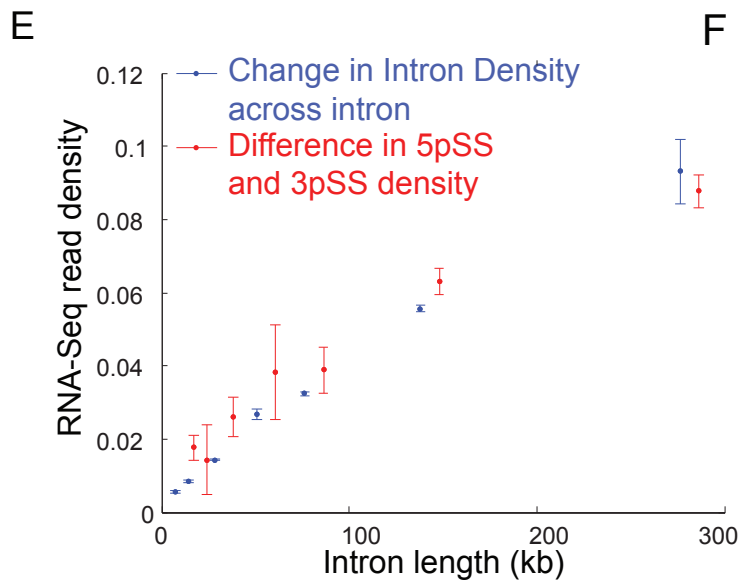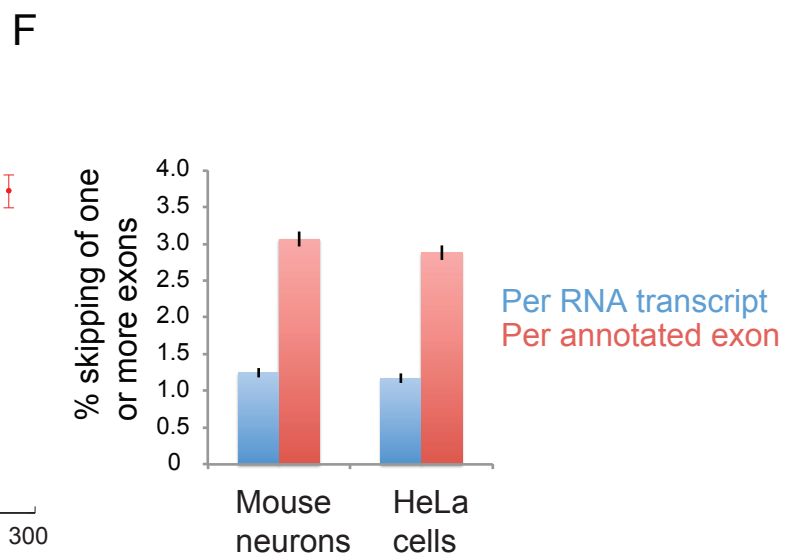

Supplement: Figure S1 — The decrease in expression across introns is not affected by excised lariat degradation or alternative splicing. (A–D) Both transcription and intron degradation could in theory contribute to the slope of intronic expression. (A) Five timelines of the intron lifecycle, each with different assumptions about the mechanisms and rates of intron degradation. Models I and IIa assume a 3′-to-5′ intronic exonuclease with a rate either equal to (I) or 10-fold faster (IIa) than that of RNA polymerase II (RNAPII). Model IIb assumes such a rapid exonuclease rate (>> 10× that of RNAPII) that it contributes negligibly to the intronic expression profile. Models IIc and III assume 5′-to-3′ exonucleases with rates ten times faster than (IIc) or equal to (III) that of RNAPII. (B) Models I, IIb, and III from A, with 5′ and 3′ splice sites (SSs) shown, emphasizing that intron degradation does not affect splice site abundances. (C) For models I, IIb, and III from A, the resulting intronic and splice site expression profiles. (D) For all models, predicted changes in the difference in expression between 5′ to 3′ ends of introns (or SSs) with increasing intron length. Only when the rate of exonucleolytic intron degradation is much faster than the rate of RNAPII are the slopes for intron ends and splice sites equal. (E) The decrease in intron density across introns and the decrease in density between 5′ and 3′ SSs are similar over a wide range of intron lengths, ruling out a significant contribution of excised lariat degradation to the intronic expression profile. Error bars represent s.e.m. from multiple introns from a single representative biological sample. (F) Approximately 1% of exon-exon splice events are between non-consecutive exons, while ∼3% of annotated exons are detectably non-consecutively spliced. We required non-consecutive events to be detected by at least two sequencing reads. Error bars are s.e.m. from 3 mouse cortical and 5 HeLa biological replicates. All data are from mou [file pone.0089673.s001.pdf]

A

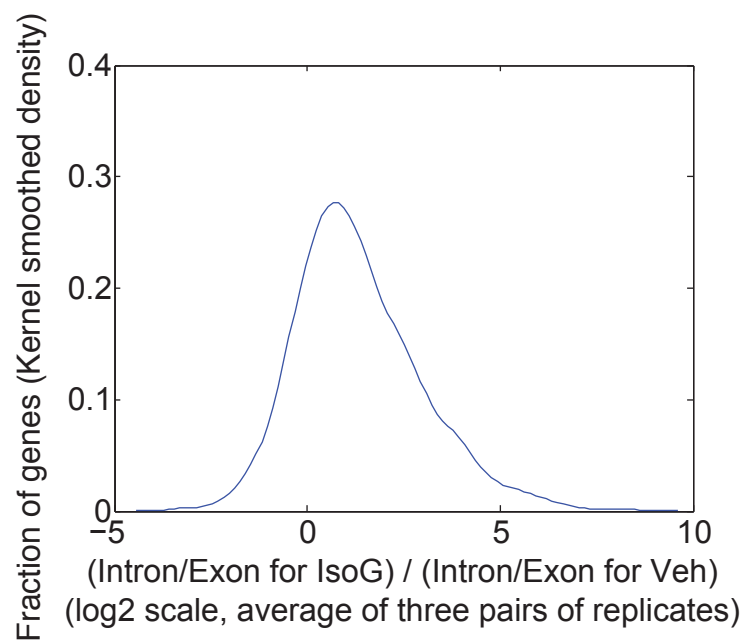

B

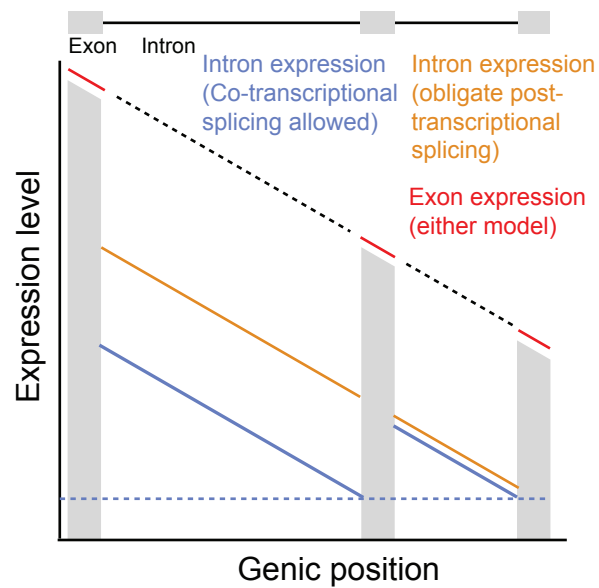

Supplement: Figure S2 — Increases in intron relative to exon expression in isoginkgetin-treated cells are not limited to a few genes, and how to interpret the sawtooth pattern of intronic expression across genes. (A) Most or all genes have increased intron to exon expression levels upon isoginkgetin treatment. The x-axis shows a ratio of ratios, indicating the increase in the intron to exon ratio upon isoginkgetin (IsoG) treatment. (B) Co-transcriptional versus obligate post-transcriptional models of splicing can be distinguished by how intron read density changes across a gene. In a model of co-transcriptional splicing in which splicing can occur at any point after the 3′SS has been transcribed (blue), the density at the 3′ end of each intron in a gene is predicted not to vary systematically along a gene. In obligate post-transcriptional splicing, in which splicing can only occur once transcription of the entire gene is complete (orange), intron density declines continuously from one intron to the next from 5′ to 3′. Decreases in intronic expression due to a uniform rate of premature termination would also be predicted to match the orange model. Regardless of the rate of splicing, the blue model holds as long as splicing is able to occur upon completion of intron transcription. (PDF) [file pone.0089673.s002.pdf]

A

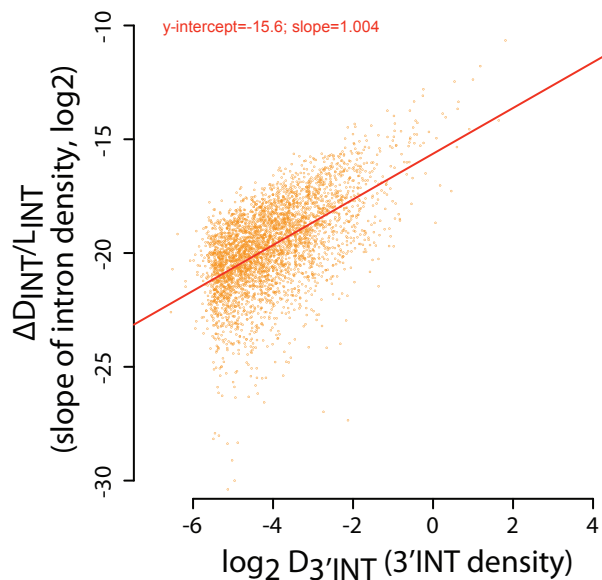

B

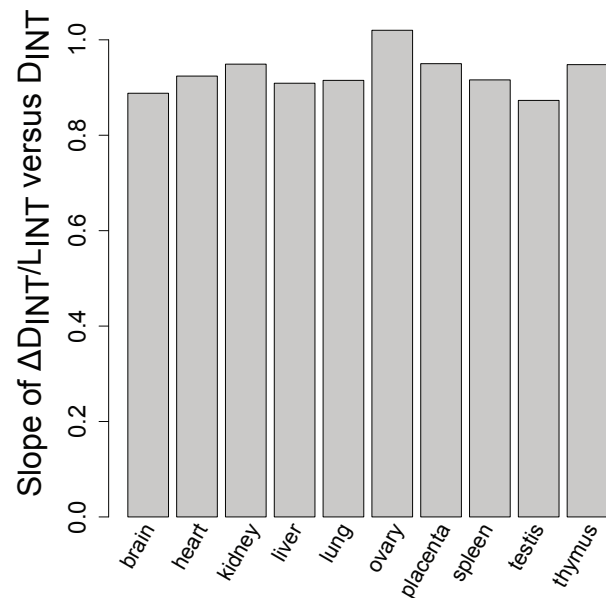

C

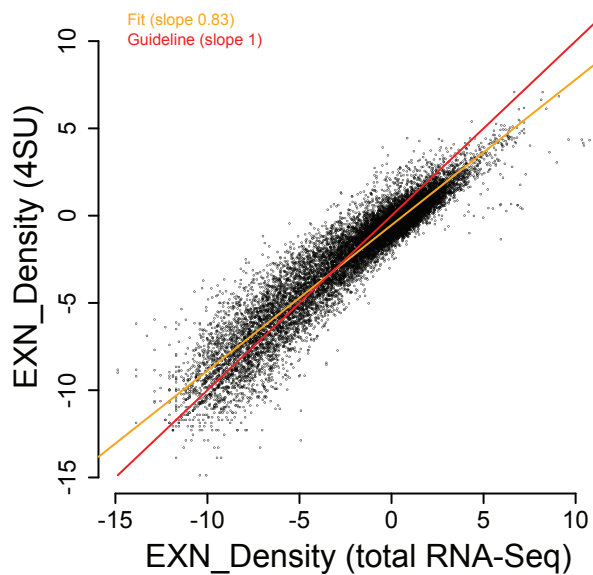

E

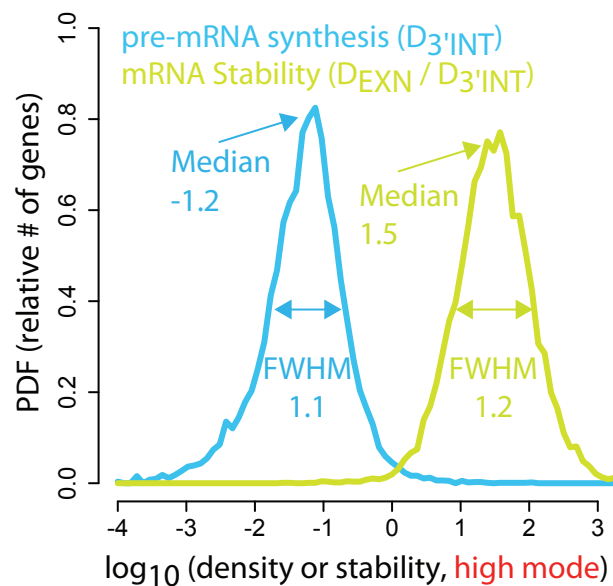

D

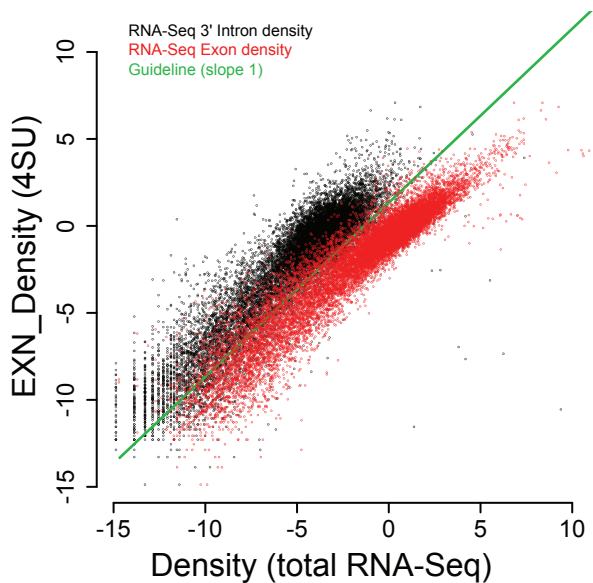

F

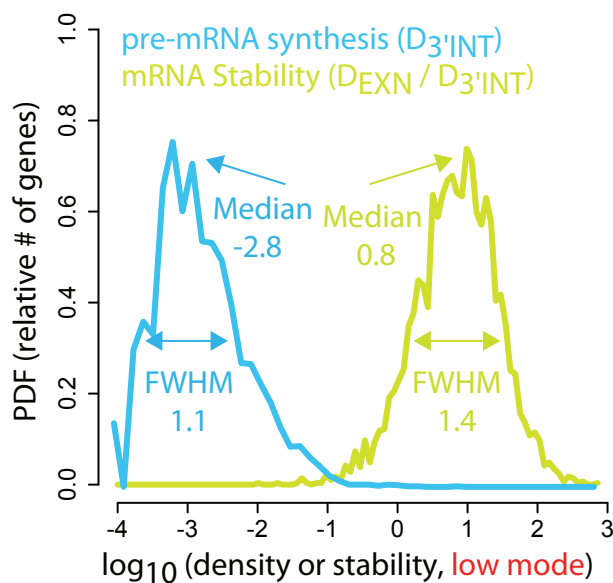

Supplement: Figure S3 — mRNA synthesis rate can be inferred from the density of reads across introns. The slope of intron density (ΔDINT/LINT) is proportional to the mRNA synthesis rate (Fig. 1A) but is difficult to measure precisely due to counting noise. Intron density (D3′INT) may be measured with greater precision, but it is proportional not only to the mRNA synthesis rate but also to the time of intron processing (T p = T 5 + T 3 + Tγ). Nonetheless, if the variability in intron processing rates were low enough relative to the variability in synthesis rates, D3′INT would be a useful proxy for synthesis rate. (A) To evaluate the utility of intron density (D3′INT) as a proxy for synthesis rate, we plotted ΔDINT/LINT versus D3′INT (total RNA-Seq, mouse neurons) and found a linear least squares log-log slope of ∼1, indicating nearly direct proportionality between ΔDINT/LINT and D3′INT. (B) The relationship shown in panel A holds across ten human tissues. (C) 4SU-Seq exon densities and total RNA-Seq exon densities are not linearly related (i.e., do not have a fit slope of 1 on a log-log scale). This result is in contrast to that for 4SU-Seq exon densities and total RNA-Seq 3′ intron densities (Fig. 4D). To make this distinction more obvious, panel (D) shows an overlay of scatterplots from Fig. 4D and panel (C). To confirm that RNA-Seq 3′ intron densities are a better indicator of mRNA synthesis rates than RNA-Seq exon densities across the full range of expression levels, we computed fits for low expressors vs high expressors (subsets of the data in panel D). Fit slopes were 0.88 (low expressors) and 0.84 (high expressors) for 4SU densities vs. RNA-Seq 3′ intron densities, and fit slopes were 0.90 (low expressors) and 0.72 (high expressors) for 4SU densities vs. RNA-Seq exon densities. We speculate that expression levels correlate more tightly with synthesis rates within the low mode of gene expression due to the ∼5-fold lower (median) mRNA stabilities in this mode (see panels E–F). (E–F) Wi [file pone.0089673.s003.pdf]

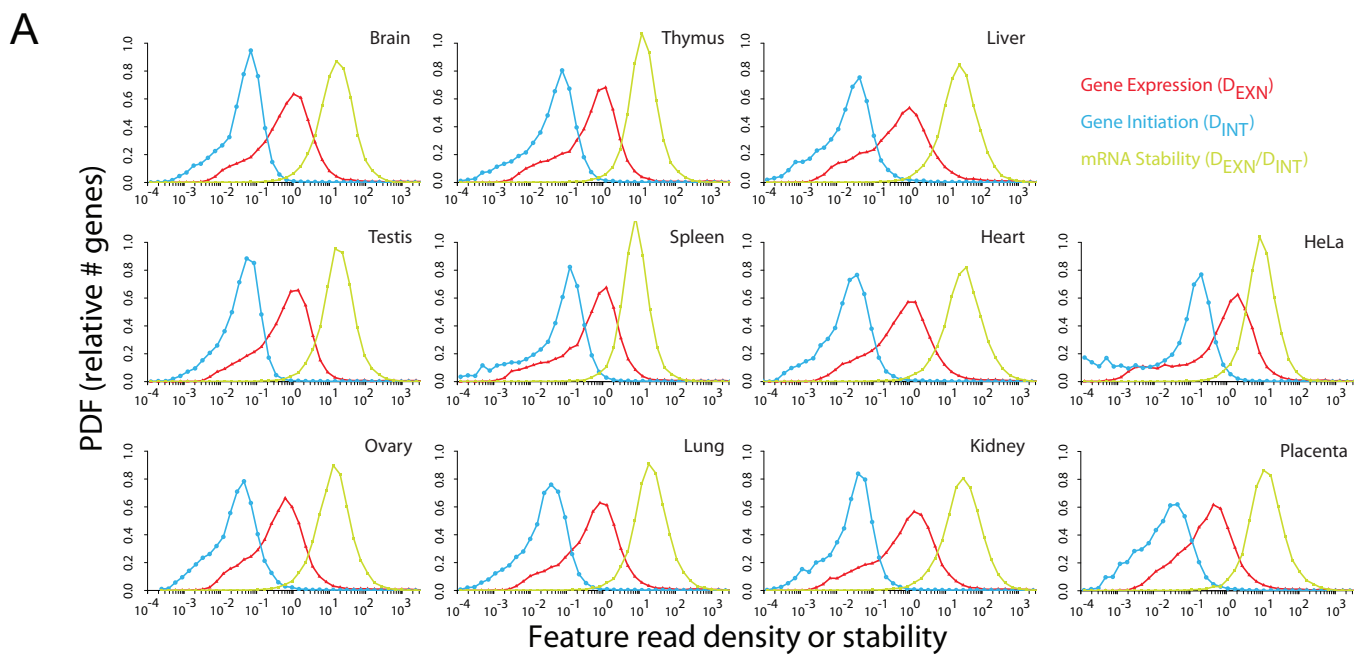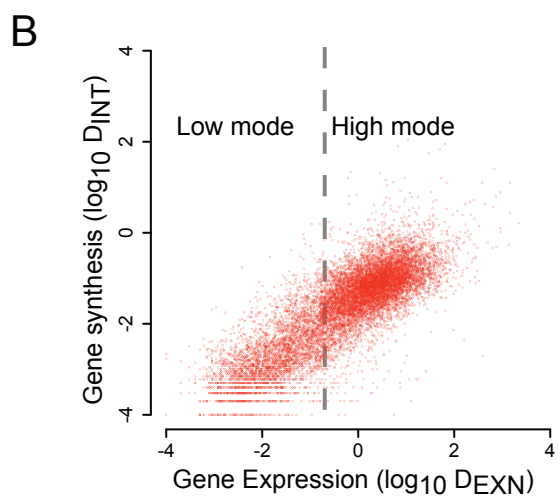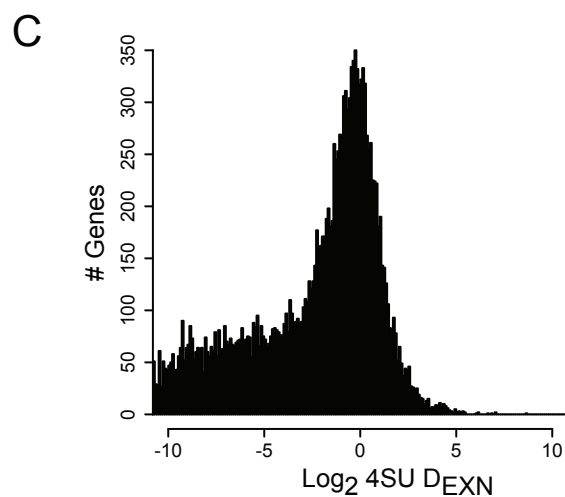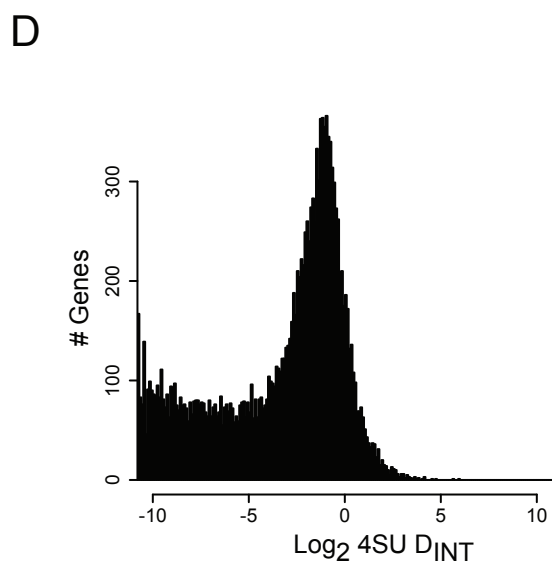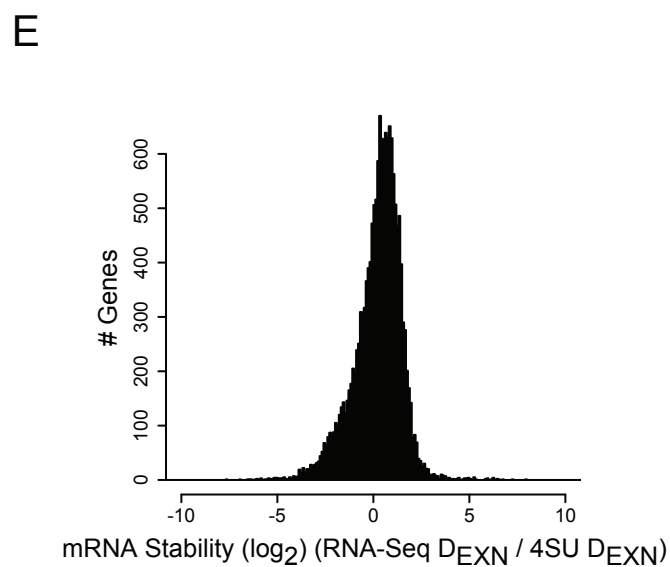

Supplement: Figure S4 — Bimodality of gene expression is driven by bimodality of mRNA synthesis. (A) Distributions of gene expression for each of ten human tissues sequenced using strand-specific total RNA-Seq on SOLiD. (B) Genes in the low or high modes of gene expression are also respectively in the low or high modes of mRNA synthesis, i.e., the low end of the x-distribution is also the low end of the y-distribution (data from mouse neurons). (C–E) As a further test of the bimodality of pre-mRNA synthesis rates but not mRNA stabilities, we confirmed that our 4SU exon (C) and intron (D) densities were bimodal. In contrast, mRNA stability was unimodal (E), even when computed using 4SU data. In (E), mRNA stability was estimated from total RNA-Seq exon densities (representing expression) divided by 4SU exon densities (representing synthesis) from the same lymphocyte cell line. (PDF) [file pone.0089673.s004.pdf]
